# Smart Wearable EEG Devices: A Review of Lightweight, Multi-Sensor Systems for Sleep and Everyday Neurophysiology

**DOI:** 10.3390/bios16070374

**Published:** 2026-07-08

**Authors:** Helena Kosnacova, Dusan Horvath, Diana Vitazkova, Erik Foltan, Michal Pecik, Erik Vavrinsky

**Affiliations:** 1 Institute of Electronics and Photonics, Faculty of Electrical Engineering and Information Technology, Slovak University of Technology, Ilkovicova 3, 81219 Bratislava, Slovakia; diana.vitazkova@stuba.sk (D.V.); erik.foltan@stuba.sk (E.F.); michal.pecik@stuba.sk (M.P.); erik.vavrinsky@stuba.sk (E.V.); 2 Advanced Technologies Research Institute, Faculty of Materials Science and Technology, Slovak University of Technology in Bratislava, Jana Bottu 25, 91724 Trnava, Slovakia; dusan_horvath@stuba.sk

**Keywords:** wearable EEG, multimodal sensors, sleep monitoring, neurophysiology, automated signal analysis, dry electrodes

## Abstract

Wearable electroencephalography (EEG) is rapidly evolving toward lightweight, user-friendly systems that enable brain monitoring in naturalistic settings. Traditional multi-channel, gel-based systems provide broad scalp coverage and high signal fidelity but are impractical for unsupervised or long-term use. This review focuses on the emerging generation of smart wearable EEG devices that are easy to wear, require minimal setup, and typically integrate additional physiological sensors such as photoplethysmography (PPG), temperature, or motion sensors. We review wearable EEG systems across four main form factors: head-worn EEG devices, smart EEG patches and tattoos, in-ear and headphone-based EEG, and glasses-integrated EEG. Head-worn systems offer broader signal coverage and support more complex applications such as sleep staging, human–machine interaction, and epilepsy monitoring. Patch-based systems are well suited to comfortable long-term monitoring, particularly in sleep-related applications. Ear-center systems provide high user comfort and stable signal acquisition from non-traditional electrode locations. Glasses-integrated devices represent an emerging option for unobtrusive daytime neurophysiology. Each category is examined in terms of sensor fusion, technical parameters, and embedded algorithms, with particular emphasis on automated signal analysis. We conclude with a discussion on current limitations, regulatory and usability challenges, and future directions toward unobtrusive, AI-powered neurotechnology for home and clinical use.

## 1. Introduction

Electroencephalography (EEG) has long been the gold standard for monitoring neural activity in clinical and research settings. Today, there is a gradual shift away from the use of traditional EEG systems that require multiple electrodes, conductive gels, extensive setup by trained personnel, and a controlled laboratory environment. There is increasing concern for participant comfort and the feasibility of long-term monitoring outside of specialized centers. Measurement limitations of standardized traditional EEG systems restrict large-scale studies of brain function during daily life and reduce their availability for applications such as home sleep monitoring or remote cognitive assessment. Polysomnography (PSG) remains the clinical reference standard for sleep stage determination but is labor-intensive and burdensome for participants, motivating the search for more user-friendly alternatives for extended physiological monitoring [1,2,3]. In sleep research, wearable EEG systems have already shown promising concordance with PSG for sleep stage determination, highlighting their potential as scalable alternatives for longitudinal sleep assessment without expert supervision.

Recent advances in sensor design, signal processing, and wearable electronics have facilitated the emergence of lightweight, comfortable EEG devices capable of unobtrusive, continuous monitoring. These technologies are increasingly being applied in areas such as sleep monitoring, neurocognitive assessment, seizure detection, and cognitive load assessment, offering the potential for long-term electrophysiological monitoring [1,4,5]. Wearable EEG systems include headbands, in-ear sensors, glasses, caps, and other compact form factors that often integrate dry or minimal contact electrodes with lightweight electronics, significantly reducing setup time and discomfort compared to conventional systems. Their key advantage lies in the ability to capture brain dynamics during naturalistic conditions, enabling repeated data collection over extended periods. This creates new opportunities for personalized health assessment and the early detection of neurological changes [1,6].

Despite these advantages, several challenges limit the broader adoption of wearable EEG devices into clinical practice and everyday health monitoring. These devices vary considerably in electrode configurations, signal quality, and the degree of validation against clinical standards such as PSG. Therefore, standardized evaluation frameworks are needed, including robust data processing methods, artifact handling procedures, and performance metrics across different physiological contexts [6]. Integration with additional physiological sensors further enhances system robustness, improves artifact identification, and supports more reliable interpretation of data. In sleep research, such multimodal approaches enable more robust long-term monitoring and open possibilities for closed-loop interventions. In brain–computer interface applications, they may improve performance under uncontrolled conditions [7,8].

The growing demand for unobtrusive, user-friendly EEG monitoring in home and everyday environments, without the limitations of conventional EEG systems, has contributed to the increasing development and adoption of wearable EEG devices. The wearable devices market is highly dynamic, with commercially available systems being continuously updated and replaced by new generations of technologies. Therefore, there is a need for an up-to-date overview of wearable EEG technologies and their applications in sleep, neurocognitive health assessment, and everyday neurophysiology domains.

The primary objective of this review is to provide a comprehensive technology-oriented overview of current smart wearable EEG systems. We summarize the main device categories and sensor integration, while also highlighting key challenges identified in the field, including signal quality and data-processing strategies, artifact management, multimodal integration, and validation requirements. Finally, we discuss the major limitations, open challenges, and future directions for the development and broader adoption of wearable EEG technologies.

## 2. Device Types, Sensor Integration, and Applications

This section presents various options for modern smart EEG systems, highlighting their technical features, placement, typical sensor combinations, and main applications (Table 1, Table 2, Table 3, Table 4, Table 5 and Table 6). Technical specifications were obtained from manufacturer documentation, whereas performance and validation data were preferentially extracted from peer-reviewed publications when available.

### 2.1. Head-Worn EEG Devices

**Figure 1:**
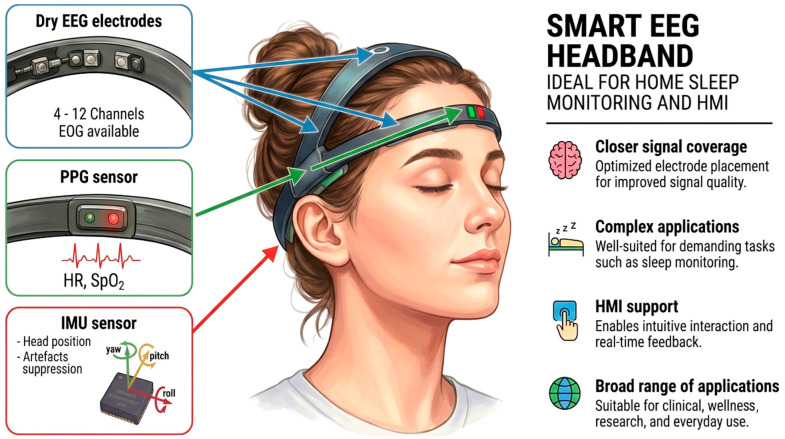
Modern head-worn EEG devices.

Head-worn EEG devices (Figure 1) represent the most advanced and widely used class of wearable systems today. Building on decades of clinical and research development of EEG caps, these systems transform conventional cable-based EEG setups into portable, user-friendly solutions suitable for long-term monitoring, ambulatory studies, and neurophysiological assessment in everyday settings. In both sleep research and real-world brain monitoring, head-worn EEG remains the dominant wearable solution due to its relatively high signal accuracy, flexible electrode placement, and compatibility with multimodal physiological sensing [1,9,10,11,12,13]. They provide access to a wide range of scalp locations, allowing the capture of spatially distributed EEG features such as slow-wave activity, sleep spindles, and regional oscillatory patterns required for sleep analysis [14,15]. Combined with complementary sensors, including inertial measurement units (IMU), photoplethysmography (PPG), and respiratory sensors, they enable comprehensive physiological monitoring beyond traditional laboratory PSG. In everyday neurophysiology, they support longitudinal monitoring of cognitive load, fatigue, stress, and neuroplasticity, while also serving as the primary sensing modality for non-invasive brain–computer interfaces (BCIs) [9,10,16,17].

This category includes reusable, helmet-like, headband-like, or cap-like structures that allow stable and repeatable electrode placement [1,6,11,18]. Depending on the application, systems range from sparse configurations (2–8 electrodes) optimized for ease of use to higher-density solutions (32 channel or more) approaching research-grade EEG performance. Most devices consist of rigid or semi-rigid bands, integrating dry or semi-dry electrodes and electronics into a single headset form factor, support wireless data transmission, and allow integration with additional sensors [1,11]. Their design enables rapid deployment without conductive gels, making them suitable for home and daily monitoring [10,19]. Although dry electrodes remain more susceptible to motion artifacts and higher skin–electrode impedance than conventional wet electrodes, advances in electrode materials and mechanical design (including conductive polymers, spring-loaded pin arrays, foam-based electrodes, and flexible conductive composites) continue to improve comfort and signal stability [20,21]. In parallel, new electrode concepts are emerging, such as hybrid dry microfluidic electrodes with controlled electrolyte release, capacitive contactless electrodes integrated into padded headbands, and biologically inspired microneedle arrays that aim to further reduce electrode-to-skin impedance while maintaining their non-invasive nature, highlighting the continued evolutionary potential of wearable EEG platforms [22]. Modern systems further incorporate low-noise amplification, real-time signal processing, adaptive power management, and long-term recording capabilities [9,23].

There is a wide range of commercial and experimental EEG head-mounted systems, reflecting various trade-offs between signal quality, portability, scalp coverage, and sensor integration. Based on mechanical design and intended application, these systems can be divided into three categories. Rigid and semi-rigid headsets prioritize signal quality and electrode stability, headband-based systems emphasize comfort and simplicity for everyday use, while cap-based devices seek to balance broader scalp coverage with wearability. Together, these categories represent different approaches to wearable EEG acquisition depending on the requirements of the intended application.

#### 2.1.1. Rigid and Semi-Rigid Headsets

The first group consists of rigid and semi-rigid head-mounted EEG systems (Table 1), worn as headsets or helmets with stably positioned electrodes. They usually offer a higher number of EEG channels, broader stable scalp coverage, and better signal-to-noise quality than lighter wearable designs, making them suitable for BCI applications, neurophysiological research, clinical monitoring, and portable sleep studies. These advantages are offset by higher weight, reduced comfort, lower robustness in real-world environments, and more demanding deployment. In neuro-research, they are widely used for applications such as motor imagery, event-related potential (ERP), P300, steady-state visually evoked potential (SSVEP), cognitive neuroscience, experimental psychophysiology, working memory research, and emotional processing, while in sleep studies they serve as portable alternatives to conventional PSG. In the following lines, we have summarized the most used devices with reference to their technical parameters and studies where they were used, if they were published.

Emotiv Epoc X [24] is a popular wireless headset device with evidence from over 20,000 published studies [25]. It has been applied in evaluating cognitive abilities [26,27], affective state monitoring [28,29], gaming [30], BCI applications [25] or assessing overall brain health and function [31,32,33]. It has also shown potential for objective pain assessment through theta activity monitoring and may be useful for sleep and neurodegenerative disease research, although it remains limited compared with clinical EEG systems [28,30,34]. The device provides 14-channel EEG acquisition, an active built-in noise-reduction mechanisms, 9-axis motion sensors, replaceable saline-based sensors allowing easy rehydration without interrupting measurements, Bluetooth 5.0 connectivity, and real-time data transmission. It supports the Emotiv software ecosystem, including EmotivPRO, Cortex API, and SDK access, with compatibility for MATLAB R2025a, Python 3.x, Unity 2023 LTS, and custom analysis pipelines. The system is GDPR, CCPA, and SOC 2 compliant.

Although Emotiv Insight is marketed as an EEG headband, it is a rigid device with a multi-arm structure with a temporal–parietal electrode configuration, placing it closer to a lightweight EEG headset than a traditional soft forehead headband device in our classification. Emotiv Insight is a portable, lightweight, and comfortable 5-channel EEG system designed for BCI applications, neurophysiological measurements, and educational or naturalistic research environments, offering rapid deployment and reliable operation with semi-dry electrodes [35]. The device is used for emotion monitoring [36], analyzing cognitive parameters [37], or BCI applications [38,39].

OpenBCI devices, including Galea and Ultracortex, support EEG recording and are suitable for research and hybrid systems. Galea enables synchronized multimodal acquisition of EEG, EMG, EOG, IMU, and optional EDA and eye tracking, whereas Ultracortex is an EEG headset that can be integrated with external biosensors. The OpenBCI Cyton platform additionally supports signals such as EMG, ECG or EDA. Both systems use dry electrodes integrated into lightweight rigid structure, enabling rapid development and Wi-Fi transmission. Galea incorporates 10 EEG electrodes in a headset designed for unobtrusive long-term wear and real-time multimodal data acquisition. Ultracortex is available with 8 or 16 dry electrodes and in three sizes, allowing setup within approximately 30 s. Its latest Mark IV version is an open-source, 3D printable headset capable of streaming data to open-source graphics software OpenBCI GUI v6.0.0-beta.1 [40,41,42]. Galea has been developed for multimodal neurophysiological research in VR/XR environments, including studies of cognition, attention, stress, emotion recognition, human–computer interaction, and hybrid BCI systems [43,44,45,46]. Ultracortex Mark IV has been widely used in BCI research, particularly for motor imagery, P300 paradigms, rehabilitation applications, assistive technologies, and EEG-controlled HMI [47,48,49].

Wearable Sensing’s DSI-24 and DSI-7 wireless systems provide signal quality suitable for use in research, neurofeedback, neuromarketing, BCIs and neuroergonomics [50,51]. They use patented dry electrodes that enable rapid deployment and have demonstrated high agreement with research-grade wet EEG systems in validation studies [52,53,54]. To improve signal quality, the devices incorporate motion and electrical artifact reduction strategies, including spring-loaded electrodes, a Faraday cage, and patented common-mode tracking technology. The DSI-24 provides full head coverage with 19 EEG electrodes, two ear clip sensors, three built-in inputs for auxiliary sensors (synchronized ECG, EMG, EOG, galvanic-skin response (GSR), respiration (RESP) and temperature (TEMP)), and an 8-bit trigger input for synchronization with external devices (eyetracking, IMU, etc.). The DSI-7 offers seven customizable EEG sensors, two ear clip sensors, and a 4-bit trigger input. Both systems feature adjustable ergonomic designs suitable for all hair types, setup time of less than 3 min (DSI-24) or 1 min (DSI-7), free recording software DSI-Streamer 8.05 supporting .csv and .edf formats, and an open API. These characteristics make them well suited for VR and real-world environments. Both DSI-24 and DSI-7 have European conformity marking (CE) intended for non-clinical use [55,56].

WR19 by ZETO, Inc. is another lightweight dry-electrode device [57] designed for rapid deployment without time-consuming preparation. The headset supports the 10–20 electrode system and records EEG and ECG signals using 19 electrodes, where the electrodes for ECG must be on the chest, 1 on the left and 1 on the right side. The device uses an additional bipolar channel which allows for the capture of synchronized ECG and has additional sensors for movement, as well as amplifiers and analog-to-digital converters built into the headset. Portability is ensured through battery operation and Wi-Fi connectivity, while signal quality has been reported to be comparable to conventional EEG [57]. The recordings are streamed in real time to the cloud for data processing. Although originally intended for clinical use [58], the Zeto WR19 EEG system is no longer available for new clinical customers as of April 2026 and is currently limited to academic and research applications [59]. The Zeto ONE device was developed for critical and emergency care, including patients in intensive care units (ICU) and seizure monitoring, often subclinical and undetectable without EEG. It can also be used in ambulances, air transport, and in home environments. It is designed for rapid deployment and seizure monitoring, accompanied by video, audio and live remote monitoring and interpretation service [60]. It is very light and comfortable, adjustable, and easy to clean with 21 soft-tip electrodes. Data is stored on the Zeto EEG cloud platform as with the Zeto WR19 and the device offers an optional expansion unit for eight auxiliary channels for a combination of EEG, EOG, ECG or EMG [61]. An additional advantage of Zeto systems is their intuitive software and ease of use, reducing the need for specialized EEG technicians. The Zeto WR19 headset has both FDA clearance and CE markings. The Zeto ONE device has FDA 510(k) clearance [62].

Advanced Brain Monitoring offers the B-Alert X-Series and StatX EEG systems, with StatX representing the FDA-approved clinical version of B-Alert. These headsets use 9 or 20 EEG electrodes requiring conductive cream to connect to the skin, with the 20-channel configuration approaching conventional EEG cap coverage, and include one selectable ECG, EOG, or EMG channel. They have been investigated as tools for assessing neurological and psychiatric conditions, BCI, neuromarketing, team neurodynamics, performance improvement, fatigue management, and safety. They come with essential acquisition software LabX and partial SDK support [63,64]. Combined with the company’s Insight platform, they support automated neurocognitive assessment for neurodegenerative and other brain disorders, enabling disease characterization, progression monitoring, and intervention evaluation [65].

Bitbrain offers the Diadem (12 channel) and Hero (9 channel) EEG headsets. Diadem covers prefrontal, frontal, parietal and occipital regions of the brain for sensing emotions and cognitive states, while Hero targets frontocentral, central and centroparietal regions for cognitive and sensorimotor monitoring. Both systems are supported by the SennsLite software platform, SennsLite v1.3.2 [66,67]. Diadem has been applied in studies of cognitive and emotional states, driver monitoring, and BCI applications, whereas Hero has been used primarily in BCI, sensorimotor, and motor neurorehabilitation research, including the decoding of movement-related EEG activity [68,69,70].

The Neuphony headset is a home-based system that provides real-time brain activity monitoring, neurofeedback training through interactive games to improve cognitive skills, and progress tracking via a mobile application [71]. The Neuphony headset has primarily been used for neurofeedback, meditation training, stress reduction, and cognitive-state monitoring. Recent studies have also evaluated its ability to record event-related potentials (P300 and MMN), resting-state EEG activity, and its agreement with research-grade EEG systems, supporting its use in cognitive and neurophysiological research [72].

Mindtooth resembles a headband but is more appropriately classified as an EEG headset due to its rigid multi-arm structure with an expandable elastic band. Unlike conventional headbands, electrodes are positioned in both frontal and parietal regions, providing broader scalp coverage and multi-regional EEG recordings. The system includes five frontal EEG sensors, three parietal sensors, reference and ground electrodes, and a magnetically attached amplifier positioned at the back of the head. Its design accommodates the use of glasses and headphones while improving fit and long-term wearing comfort [73]. The device provides approximately 3 h of continuous operation and is intended for professional, non-medical applications. It has been used to monitor workload, stress, attention, alertness, collaboration, and team dynamics in real time across a range of operational environments, including pilot training, driving simulation, industrial workplaces, healthcare, and education [74,75,76,77,78,79,80]. Studies in pilots and drivers demonstrated its ability to provide objective neurometric indicators of mental state, workload, and fatigue, supporting training evaluation, driver-state monitoring, and road safety applications. Additional research has investigated team collaboration among pilots and surgeons, workload-adaptive interventions in industrial workers [81], neurophysiological responses to artworks and cultural heritage objects [82], and the assessment of educational processes [83].

Neurosity is an EEG-capable device primarily marketed for cognitive-state monitoring, attention tracking, and productivity-related applications. The Neurosity SDK (Software development kit) can be used to develop custom neurotechnology applications, and users may participate in the Neurosity Developer Program to create and evaluate new solutions [84,85]. Neurosity devices have been used for cognitive-state monitoring, attention tracking, fatigue assessment, passive brain–computer interfaces, and adaptive BCI applications, particularly in real-world and augmented reality environments [86].

Mindwave Mobile 2 is a one-electrode EEG device that provides neurofeedback, cognitive data, meditation training, focus, and overall custom app creation with an open SDK. Although it resembles a headband, its rigid structure and extended electrode configuration place it within the headset category in our classification [87]. MindWave Mobile 2 has been widely used in neurofeedback, meditation training, attention monitoring, educational research, cognitive assessment, and low-cost BCI studies. It has also been employed in investigations of mindfulness training and attention enhancement using wearable EEG technologies [88,89,90,91,92].

**Table 1:** Rigid and semi-rigid head-mounted EEG.

Device	EEG ^1^ Channels/Electrode Type	Sampling Rate/Resolution	Other Sensors	Applications	Other Specification	Ref.
Emotiv EPOC X	14/Saline soaked felt pads	128/256 Hz, 14/16-bit	IMU ^2^	BCI ^3^, Mental Commands, Gaming, Cognitive studies, Facial Expressions	BPF ^4^ 0.16–43 Hz, Notch filter, Dynamic range 8400 μV (p-p ^5^), CMS/DRL ^6^ references at P3/P4, BLE ^7^ or 2.4 GHz USB ^8^ receiver, 595 mAh Li-Po ^9^ battery, 9 h operating time, Weight 170 g, Dimension 9 × 15 × 15 cm, GDPR ^10^, CCPA ^11^ and SOC2 ^12^ compliant	[24]
Emotiv Insight	5/Semi-dry polymer	128 Hz, 16-bit (0.1275 μV)	IMU	BCI, Cognitive studies, Facial Expressions	BPF 0.5–43 Hz, Notch filter, Digital 5th Sinc filter, Dynamic range 8400 μV (pp), BLE, 480 mAh Li-Po battery, 20 h operating time	[35]
OpenBCI Ultracortex Mark IV	8 or 16/Dry Ag/AgCl	250 Hz	Optional EMG ^13^, EOG ^14^, IMU, PPG ^15^	BCI, EEG, Neurofeedback, Cognitive studies	3 cap sizes, 500 mAh, Li-ion ^16^ battery GUI ^17^ OpenBCI	[41]
OpenBCI Galea	10/Dry	250 Hz	VR ^18^, Eye tracking EOG, 6× EMG, IMU, PPG (ear), EDA ^19^	BCI, EEG, VR, Cognitive studies, Facial Expressions	Removable HMD ^20^, Wi-Fi ^21^, 4× ExG ^22^ channels	[42]
Wearable Sensing DSI-24/DSI-7	24 or 7/Dry	300 Hz (600 Hz), 16-bit (0.317 μV)	Optional ECG ^23^, EMG, EOG, GSR ^24^, RESP ^25^, TEMP ^26^, IMU	BCI, Neurofeedback	2 cap sizes, BPF 0.003–150 Hz, Input impedance 47 GΩ, CMRR ^27^ 120 dB, Evoked potentials, Signal Quality Monitoring, Raw and filtered data, CSV ^28^ and EDF ^29^ format, TCP/IP ^30^ BT ^31^, 24 h operating time, CE ^32^ for non-clinical use	[55,56]
Zeto WR-19	19/Dry (AgCl coated plastic)	500 Hz, 24-bit (0.044 μV)	Differential ECG, ACC ^33^, Trigger	Clinical use, ambulatory monitoring	Signal range ± 300 mV, Leakage current < 1 pA, Noise 1 μV, CMRR 120 dB, Input impedance 1000 GΩ, Input capacitance 10 pF, Wi-Fi, Li-ion battery, 6 h operating time, Dimension 214 × 274 × 144 mm, Weight 800 g, FDA ^34^ cleared	[59]
Zeto ONE	21/ (Soft-tip)	500 Hz	Optional EEG, ECG, EOG, EMG, ACC	Seizures monitoring on ICU ^33^, Ambulatory monitoring	High-definition video, 8 auxiliary biopotential channels, Light stimulation, Complete Zeto EEG cloud, Weight 400 g, CE, FDA cleared	[61]
B-Alert X/Stat X	9 or 20/ Wet (Ag/AgCl)	256 Hz, 16-bit	ACC (12-bit)	Biomarkers, BCI, Neuro-research	BPF 0.1–67 Hz, Dynamic Range ± 1 µV, Input impedance 500 MΩ, CMRR 115 dB, Noise 3 μV (PP), BT, 650 mAh Li-ion battery, 12 h operating time, EDF format, SDK ^35^, On-line impedance monitoring, Unit dimensions 6.9 × 4.8 × 2 cm, Weight 57 g, FDA cleared	[63,64]
BrainBit Hero/Diamond	9 or 12/Dry	256 Hz, 24 bit	IMU	Cognitive state, Emotions, Sensorimotor states	Diamond: BT, Input range ± 100 mV, Noise 1 µV RMS, Li-Po battery, 8 h operating time Hero: BT, μSD card, Li-Po battery, 3 h operating time, CSV, EDF formats, CE	[66,67]
Neuphony	6/Dry	250 Hz, 24 bits (22.35 nV)	ACC	Neurofeedback	Fp1, Fp2, F3, F4, Fz, T3, T4, Pz electrodes, Polycarbonate with Ag/AgCl coating, BPF 1–45 Hz, Dynamic Range 375 mV (p-p), BLE, 8 h operating time, CE	[71]
Mindtooth	8/Wet (Ag/AgCl)	125–500 Hz, 24-bit	-	Workload, Stress, Attention	BPF 0.1–33 Hz (125 Hz), 0.1–66 Hz (250 Hz), 0.1–131 Hz (500 Hz), 52–60 cm head circumference, BLE, 240 mAh Li-ion battery, 3 h operating time, Dimensions 53 × 53 × 15 mm, Weight 200 g, CE and UKCA ^36^, CSV format	[73]
Neurosity Crown	8/Dry (Ag/AgCl)	256 Hz	IMU, 2× Haptic motors	EEG, Increasing productivity	Noise 0.25 µV RMS ^37^, 1.8 GHz N3 quad-core processor, 8 GB flash storage, 1 GB RAM, BLE, NFC ^38^, Wi-Fi, Cloud, Li-ion battery, 3 h operating time, Dimensions 16.5 × 15.9 × 8.3 cm, Weight 228 g	[84]
MindWave Mobile 2	1/Dry	512 Hz, 12 bit	ECG	BCI, Neurofeedback, Meditation,	BPF 3–100 Hz, EEG/ECG signal quality analysis, BLE, 8 h operating time, Dimensions 225 × 155 × 92 mm, Weight 90 g	[87]

^1^ Electroencephalography. ^2^ Inertial measurement unit. ^3^ Brain–computer interface. ^4^ Band-pass filter. ^5^ Peak-to-peak. ^6^ Common mode sense/driven right leg. ^7^ Bluetooth low energy. ^8^ Universal serial bus. ^9^ Lithium-polymer battery. ^10^ General data protection regulation. ^11^ California consumer privacy act. ^12^ Service organization control 2. ^13^ Electromyography. ^14^ Electrooculography. ^15^ Photoplethysmography. ^16^ Lithium-ion battery. ^17^ Graphical user interface. ^18^ Virtual reality. ^19^ Electrodermal activity. ^20^ Head-mounted display. ^21^ Wireless local area network. ^22^ Generic electrophysiological or biopotential channels. ^23^ Electrocardiography. ^24^ Galvanic skin response. ^25^ Respiration. ^26^ Temperature. ^27^ Common-mode rejection ratio. ^28^ Comma-separated values. ^29^ European Data Format/European Data Format Plus. ^30^ Transmission Control Protocol/Internet Protocol. ^31^ Bluetooth. ^32^ European conformity. ^33^ Accelerometer. ^34^ Food and Drug Administration. ^35^ Software development kit. ^36^ UK conformity assessed. ^37^ Root mean square. ^38^ Near-field communication.

#### 2.1.2. Headband EEG Devices

Headband-based wearable EEG systems (Table 2) are currently among the most widely used EEG monitoring solutions outside the laboratory. Unlike rigid and semi-rigid headsets, they prioritize comfort even for long-term wear or sleep, simplicity, and rapid deployment. Typically worn around the forehead and temples, they use dry or semi-dry electrodes in frontal or prefrontal regions and acquire 1–4 EEG channels, making them suitable for long-term home and ambulatory monitoring. Their lightweight design makes them particularly suitable for undisturbed sleep research and monitoring, sleep stage determination, cognitive load assessment, attention tracking, and stress or fatigue detection.

Additional sensors such as PPG and IMU modules are often integrated to improve physiological context and artifact handling. Their main limitation is reduced spatial coverage (mainly posterior regions) and lower EEG resolution compared with headset-based systems; however, they provide sufficient information for many monitoring applications and simpler BCI approaches. This category includes well-known devices such as Muse, Dreem, and others.

Muse devices are among the most widely used wearable EEG systems. Muse 2 ATHENA is a textile headband with seven EEG sensors, PPG-based heart rate (HR) monitoring, and motion sensors for breathing and movement assessment. The device is easy to clean and handle. It complies with FCC, UL and CE standards [93,94,95,96,97,98] and has been applied in sleep monitoring, meditation, neurofeedback, emotion recognition, cognitive-state assessment, and EEG-based mental health research. It creates a sleep laboratory in the warmth of home with a comprehensive understanding of sleep patterns and helps improve sleep habits. Compared with Muse 2, it additionally supports sleep staging and sleep-quality assessment, providing home-based sleep monitoring with reported agreement to laboratory measurements and is from softer adaptive fabric suitable for uninterrupted sleep. Muse 2 focuses primarily on concentration training, stress reduction, and monitoring of physiological parameters such as breathing, body position, and HR. It does not include functional near-infrared spectroscopy (fNIRS) for monitoring cerebral blood flow and oxygenation. Despite its limited number of channels (e.g., 4), studies have demonstrated its utility for detecting basic EEG rhythms, emotional states, attention, meditation, and neurofeedback applications with tools to improve cognitive abilities [99,100,101].

The Dreem 3S headband represents a valuable research tool, especially for sleep monitoring and the assessment of brain activity. This FDA-cleared device [102] combines algorithms and AI for improved characterization and personalization. By providing clinically validated sleep-stage data comparable to PSG [103,104], it enables experiments and clinical trials to be conducted in the home environment [105]. The device is comfortable, does not disturb sleep, and can be paired with the Beacon Pal application, which converts data into useful information. Data security complies with SOC 2 Type 2 certification, US DPF registration (aligned with GDPR) and GCP [106].

BrainCo offers three headsets for neurofeedback, cognitive training and BCI applications: OxyZen, Easleep and FocusCalm. OxyZen is designed for neurofeedback, meditation and sleep, measuring not only EEG but also PPG to determine HR and blood oxygen [107]. Easleep is intended primarily for sleep monitoring and improvement, offering neurofeedback through an eye-mask form factor. It is designed not only for nighttime sleep but also to facilitate relaxation during short naps at work or while traveling [108]. FocusCalm is intended for concentration enhancement and stress reduction through neurofeedback and a variety of game-based and meditation exercises [109]. However, relatively few studies have been conducted using this device. It is primarily intended for monitoring brain activity and applications related to sleep improvement, concentration enhancement, relaxation, and meditation. For example, FocusCalm has been used to monitor students’ attention and concentration during online learning, suggesting that EEG-based monitoring may offer a more objective assessment of cognitive engagement than questionnaire-based or webcam-based methods [110].

The versatile OpenBCI headband can be combined with the 4-channel or 8-channel OpenBCI Ganglion or Cyton board. Using Ag/AgCl electrodes, it provides a simple and flexible platform for BCI development, emotion recognition, mental-state monitoring, education, and neuroscience research [111,112].

Advanced Brain Monitoring is an innovative neuro-diagnostic company offering a range of sleep monitoring and advanced brain monitoring devices. The Sleep Profiler™ EEG device provides PSG-level sleep monitoring and records multiple physiological signals, including EOG, EMG, ECG, HR, head position, head movement, and quantitative snoring, supporting sleep stage assessment and broader sleep analysis. It is suitable and used in ICU patients and supports the automatic generation of summaries of medical history, medications, patient information, and comparisons between sleep diaries and objective sleep quality measurements. It is also used in home somnography, research, and the diagnosis of neurodegenerative and other brain diseases [113,114]. All signals are wirelessly transmitted to a tablet with real-time evaluation. The ability to record for up to 30 h between battery charges is advantageous for long-term monitoring [115]. The PSG2 Sleep Profiler combines the Sleep Profiler with cardiorespiratory signals for PSG. It is a 13-channel system with EEG (three sensors), EOG, EMG, peripheral oxygen saturation (SpO_2_), nasal pressure/airflow, respiratory effort at the chest and abdomen, pulse rate at the forehead and fingers, head movement and position, and quantitative snoring. It is the only device in this category specifically designed for integrated sleep apnea monitoring [116]. It is suitable for clinical examinations as well as home use and research [117].

The BrainBit headband device is flexible and designed for daily real-time recording. It can record various bioelectrical activities with four dry electrodes, an integrated electronic module, and a removable battery. It has been used in BCI, meditation, neurofeedback, health monitoring, sports performance monitoring, traffic safety research, VR, augmented reality (AR) applications, sleep pattern monitoring, and cognitive-state assessment. The device includes a free SDK and is intended for non-medical applications [118].

The Unicorn BCI CORE-4 from g.tec is an EEG headband designed primarily for BCI and high-quality reliable data collection. Operation is quick and easy thanks to dry electrodes that can be simply connected and used. The g.Pype platform offers a free Python SDK for creating BCI applications [119,120].

The Neeuro SenzeBand 2 is a non-invasive neurofeedback device that uses algorithms and machine learning. It analyzes measured data to assess an individual’s mental state and offers the NeeuroOS system for attention, meditation and mental load monitoring. Applications include research, athlete training, neuromarketing, and cognitive assessment [121].

The BrainAccess HALO is an ultra-lightweight headband for everyday use with an integrated amplifier and battery designed for research, education, wellness, and non-clinical neurotechnology applications. It has been employed in BCI development, cognitive-state monitoring, neurofeedback, and neuroscience research [122,123].

The eight-channel EEG device Air from Bitbrain covers prefrontal and occipital regions and is optimized for estimating basic cognitive and emotional states. It has been used in cognitive neuroscience, emotion recognition, neuroergonomics, and BCI research [124]. Bitbrain also offers fully soft headbands with textile electrodes, including the Ikon 5ch EEG and Ikon Sleep with two pre-gel forehead sensors for sleep monitoring. These devices prioritize comfort and ease of use for sleep monitoring, cognitive assessment, and research applications while complying with standards for the design of medical devices [125,126].

**Table 2:** Headband-based EEG.

Device	EEG ^1^ Channels/Electrode Type	Sampling Rate/Resolution	Other Sensors	Applications	Other Specification	Ref.
Muse S ATHENA	7/Dry (20% Ag fabric)	256 Hz, 20-bit	PPG ^2^, fNIRS ^3^ (64 Hz), SpO_2_ ^4^, ACC ^5^, GYRO ^6^	Sleep monitoring, Neurofeedback	43–63 cm head circumference, BLE ^7^, 150 mAh Li-ion ^8^ battery, 10 h operating time (3 h charging), Weight 41 g	[99]
Muse 2	4/Dry (Au) + 2 Aux ^9^	256 Hz, 16-bit	PPG, RESP ^10^, ACC, GYRO	BCI, Neurofeedback, Cognitive training	BT ^11^, Li-ion battery, 5 h operating time (3 h charging), Weight 38.5 g, CE ^12^	[101]
Dreem 3S	5/Dry	250 Hz	ACC, RESP	Sleep studies, Clinical trials, Sleep staging	BPF ^13^ 0.03–35 Hz, 8 h operating time, Raw data, EDF format, FDA ^14^ cleared (510k), Sleep staging SMAPE ^15^ < 10%, MCC ^16^ 0.77	[102,103]
OxyZen	3/Dry	-	PPG, SpO_2_	Neurofeedback, Sleep monitoring	BT, 8 h operating time, Weight 57 g,	[108]
Easleep	4/Dry	-	-	Sleep monitoring	Cranial electrotherapy stimulation, BT, 52–62 cm head circumference, Data processed locally, 10 h operating time, Weight 68 g	[109]
FocusCalm	3/Dry	250 Hz	IMU ^17^	Neurofeedback, Cognitive training	BT, 8 h operating time, Weight 105 g, CE	[110]
OpenBCI Headband	8/Dry (Ag/AgCl)	200/250 Hz	Additional EOG ^18^, EMG ^19^	BCI ^20^, Emotions	Cyton board (8 channels) or Ganglion board (4 channels, LPF ^21^ 160 Hz)	[111]
Sleep Profiler	3/Dry	256 Hz	EOG, EMG, ECG ^22^, HR ^23^, ACC, Snoring	Sleep monitoring	Frontal electrodes, Auto-staging, Arousals detection, Sleep, REM ^24^ and N3 ^25^ latency, 4 GB RAM, 30 h operating time, BT, Dimensions 7 × 4.5 × 1.75 cm, Weight 71 g, FDA	[116]
PSG2	3/Dry	256 Hz, 16-bit (0.03 μV)	EOG, EMG, PPG, SpO_2_, Nasal pressure, RESP, ACC, Snoring	Sleep monitoring	BPF 0.1–67 Hz, PPG (16-bit), ACC (12-bit, 10 Hz), Microphone (16-bit), Noise 3.7 μVpp, Airflow range ± 2 cm H_2_O, SpO_2_ accuracy ± 2%, 30 h operating time, Weight 388 g, FDA, CE	[118]
BrainBit Headband	4/Dry (Au plated)	250 Hz	EMG, EOG	BCI, Neurofeedback, VR, AR ^26^, Sleep patterns	O1, O2, T3, T4 electrodes, EEG range ± 0.4 V, BPF 0–100 Hz, BLE, 12 h operating time, FCC ^27^, CE certificates, SDK ^28^, Artifacts detection algorithms, CSV, EDF+ formats	[127]
Unicorn BCI Core-4 Headband	4/Dry	250 Hz, 24-bit		BCI, research	BPF 0–15.92 kHz, Wireless EEG recording, Sensitivity ± 150 mV, Input Impedance 1 GΩ, Li-Po battery, 6 h operating time, Weight 76 g, Safety Class II, BT, CE certified	[120]
Neeuro SenzeBand 2	7/Dry	250 Hz, 24 bit (0.02235μV)	PPG IMU	Neurofeedback	Fp1, Fp2, T3, T4, Fpz electrodes, 8.5—25 cm head circumference, Input range ± 187.5 mVpp, BT, 320 mAh Li-Po ^29^ battery, 6 h operating time, Dimensions 17.9 × 15.1 × 3.1 cm, Weight 88 g, FDA, CE	[128]
BrainAccess Halo	4	250–500 Hz, 24 bit		Research, Educational, Wellness, BCI	Fp1, Fp2, O1, O2 electrodes, 53–58 cm head circumference, Input voltage range 450 mV, BLE, 400 mAh Li-Po battery, 7 h operating time, Weight 70 g	[123]
BitBrain Air	8/Dry	256 Hz, 24 bit	IMU	Cognitive state and emotions, BCI	Fp1, Fp2, AF7, AF8, PO7, PO8, O1, O2 electrodes, BPF 0.5–30 Hz, Input range ± 100 mV, Noise < 1 µV RMS, Input impedance 50 GΩ, BT, CSV, EDF formats, Li-Po battery, 6 h operating time, SDK, Dimensions 51 × 71 × 30 mm, Weight 82 g, CE	[125]
BitBrain Ikon/Ikon sleep	5 Dry (Textile)/2 Wet	256 Hz, 24 bit	IMU/IMU, PPG	Research, Sleep	BitBrain: Fp2, Af7, Af8, A1, A2, Fpz, FP1 electrodes, 52—70 cm head circumference, Ikon sleep: M2, M1 electrodes, LPF 40 Hz, Input range ± 100 mV, Noise < 1 µV RMS ^30^, CMRR ^31^ 100 dB, Input impedance 50 GΩ, BLE, CSV, EDF formats, SDK, Li-Po battery, 9 h operating time, Dimensions 79 × 42 × 35 mm, Weight 65 g, CE and FCC certified	[129]

^1^ Electroencephalography. ^2^ Photoplethysmography. ^3^ Functional near-infrared spectroscopy. ^4^ Peripheral capillary oxygen saturation. ^5^ Accelerometer. ^6^ Gyroscope. ^7^ Bluetooth low energy. ^8^ Lithium-ion. ^9^ Auxiliary. ^10^ Respiration. ^11^ Bluetooth. ^12^ European conformity. ^13^ Band-pass filter. ^14^ Food and Drug Administration. ^15^ Symmetric mean absolute percentage error. ^16^ Matthews correlation coefficient. ^17^ Inertial measurement unit. ^18^ Electrooculography. ^19^ Electromyography. ^20^ Brain–computer interface. ^21^ Low-pass filter. ^22^ Electrocardiography. ^23^ Heart rate. ^24^ Rapid eye movement. ^25^ Stage 3 Non-REM sleep. ^26^ Augmented reality. ^27^ Federal Communications Commission. ^28^ Software development kit. ^29^ Lithium-polymer. ^30^ Root mean square. ^31^ Common-mode rejection ratio.

#### 2.1.3. Cap-Based EEG Devices

The third category includes cap-based lightweight EEG systems (Table 3), which combine the comfort and wearability of headbands with broader scalp coverage and improved signal acquisition. These elastic textile caps offer fast setup and integrate dry or semi-dry electrodes through hair-compatible designs that do not require conductive gels. Compared with headbands, they provide more comprehensive EEG recordings, making them suitable for detailed sleep monitoring, advanced BCI applications, and neurophysiological research outside the laboratory.

The main trade-off is the balance between comfort and electrode density, as increased scalp coverage may require a more complex setup and reduce long-term wearability. Nevertheless, modern textile materials are soft and washable, and wireless designs enable relatively rapid deployment and long-term use in ambulatory and home environments. Although signal quality may still be affected by movement or electrode pressure, these systems generally provide a practical compromise between wearability and EEG performance [130,131].

The g.Nautilus is a fully wireless soft-cap EEG system designed for research and home environments. Available with 32 channels and hybrid dry/wet electrode configuration, it supports incorporation of additional sensors such as EMG, EOG, and IMU. The system has been widely used in BCI, cognitive neuroscience, neuroergonomics, motor-imagery research, and mobile EEG studies, demonstrating the ability to acquire reliable EEG during movement. Although used less frequently for sleep monitoring, it meets all necessary parameters. The combination with eye tracking (Tobii Pro Glasses 3) enables a synchronized solution for understanding the connections between brain activity, visual engagement, and behavior, providing deeper insight into cognitive processes such as attention, perception, visual behavior, and decision-making [132,133].

The Emotiv Epoc Flex is a 32-channel EEG cap capturing brain activity across the scalp, with optional ECG and EOG acquisition. The system provides reproducible recordings with integrated noise suppression through EmotivPRO v3.0 software. It supports saline or gel electrodes, with the latter being more suitable for longer measurements. Validation studies have shown that the saline version can achieve research-grade EEG quality [134]. Applications include cognitive neuroscience, BCI, neurophysiological monitoring, emotion recognition, and AI-assisted robotic control, where the gel-based version has been integrated with mobile robot control systems [135].

OpenBCI offers an EEG cap compatible with Cyton (8 channels) and CytonDaisy (16 channels) boards. These comfortable, easy-to-clean caps feature 19 electrodes and an adjustable chin strap to prevent unwanted movement and associated artifacts. It is a scientifically proven research tool, for example, in sleep monitoring. They have been used in sleep monitoring, BCI development, neuroscience education, cognitive research, and open source neurotechnology projects [124,136,137].

The Unicorn Hybrid Black from g.tec is a versatile device for brain activity analysis, research, and BCI applications. It has been widely applied in motor-imagery BCIs, neurofeedback, assistive technologies, cognitive neuroscience, rehabilitation research, and educational BCI development. The Unicorn Suite 1.24 software facilitates rapid implementation of new BCI applications [138,139,140,141,142].

The CGX Quick dry-electrode caps are available in 20 or 32 electrode versions following the international 10–20 system. The Quick-32r and Quick-20v are suitable for research, and the Quick-20m is FDA-cleared for medical applications. It requires less than five minutes, and two auxiliary inputs allow synchronization with additional sensors. These systems have been used in cognitive neuroscience, ERP studies, neuroergonomics, BCI research, and clinical EEG monitoring [143,144,145].

BrainBit Flex is a four-channel EEG offering a free SDK designed for research, education, training, wellness, and neurofeedback applications. Related systems include Flex8 and DragonEEG. DragonEEG has 21 electrodes and is intended for qEEG brain mapping, advanced research, and professional neurotechnology development, while Flex8 targets neuroscience studies, neurofeedback training, education, and product development. These systems also support acquisition of auxiliary biosignals for artifact control during experiments [146,147,148].

Enobio^®^ systems are medically certified devices available with 8, 20 or 32 electrodes and either dry or wet electrodes. They are used in clinics, neuroscience research, BCI development, cognitive assessment, and neurotechnology application development through available SDKs. Neuroelectrics also offers Starstim^®^ tES neurostimulation systems, which combines EEG monitoring with transcranial electrical stimulation and has been extensively used in research on pain, epilepsy, Alzheimer’s disease, stroke rehabilitation, depression and addictive disorders [149,150].

In summary, head-worn EEG remains the most established wearable category for measuring brain waves due to its balance of signal quality, scalp coverage, and compatibility with multimodal sensing. New research is pushing the capabilities of these devices to new levels. Although challenges related to motion artifacts, electrode–skin contact variability, and comfort remain, head-worn systems continue to serve as the reference standard against which emerging wearable EEG form factors are evaluated. Recent validation studies and meta-analyses indicate moderate to high agreement with PSG and conventional EEG, although performance varies across devices and applications.

Other wearable EEG paradigms, such as adhesive patches and epidermal systems, are discussed separately in the following sections, as they introduce fundamentally different constraints in terms of electrode–skin coupling, spatial coverage and system integration, which directly affect the achievable signal quality and application areas.

**Table 3:** Cap-based lightweight EEG.

Device	EEG ^1^ Channels/Electrode Type	Sampling Rate/Resolution	Other Sensors	Applications	Other Specification	Ref.
g.tec g.Nautilus	8/16/19/32/Dry or wet	250/500 Hz	Optional ECG ^2^, EMG ^3^, EOG ^4^, fNIRS ^5^, ACC ^6^, 8× Trigger	Research, BCI ^7^, Neuroscience	Selectable sensitivity ± 2.25 V to ± 187.5 mV, Noise 0.6 µV RMS ^8^, Input impedance 1 GΩ, BT ^9^, Li-ion battery, 10 h operating time, Dimensions 77 × 101 × 32 mm, Weight 143 g, FDA ^10^, CE ^11^ certified	[132,133]
Emotiv Flex	32/Gel or saline	128/256 Hz, 16-bit (0.51 μV)	Time-locked EEG, ECG, EMG, EOG, IMU ^12^	BCI research, Sleep monitoring, Mental commands	BPF ^13^ 0.16–43 Hz, Notch filter, Dynamic range 8400 μV(pp), Proprietary 2.4 GHz USB receiver, 3 cap sizes, BT, CMS/DRL ^14^ configurable, Dual ADC ^15^, 595 mAh Li-Po battery, 6 h operating time	[151]
OpenBCI EEG cap	19/Gel or saline	125/250 Hz (0.298 μV)	EOG, EMG, ACC	Ambulatory EEG, Sleep monitoring	3 cap sizes, Cyton 8 channel or CytonDaisy 16-channel board, ADS1299 AFE ^16^, BLE ^17^	[152,153]
GTec Unicorn	8/Dry or wet	250 Hz, 24 bit	ACC, GYRO ^18^	Research, BCI, Neurofeedback	Sensitivity ± 750 mV, Input Impedance 1 GΩ, BT, Li-Po battery, 3 h operating time, Weight 56 g, CE certified	[138,140]
Cognionics Quick	20 or 32/Dry	500 Hz, 24 bit	ACC	Research, Neurofeedback	Active Electrodes, LPF ^19^ 131 Hz, 52–62 cm head circumference, Noise < 1.0 μV RMS, BLE, EDF or CSV formats, 8 h operating time, Dimensions 20 × 18 × 19 cm, Weight 646 g, FDA certified	[143,144,145]
ANT Neuro Eego sports/Mylab cap	8–64/Wet/Dry	2048 Hz 24 bit	IMU	Mobile neuroscience	Input Impedance > 1 GΩ, Trigger Input: 8-bit TTL, USB-C 3.0, 5 h operating time, Dimensions 20.6 × 15.7 × 25 cm, Weight 600 g, FDA, CE	[154]
BrainBit Flex/Flex8	4 or 8/Dry or wet	250 Hz		Research, Neurofeedback	Flex 8: Free 10–20 electrode placement, BLE, Open SDK ^20^, BrainBit Flex: O1, O2, T3, T4 electrodes, 2 caps sizes, Input range ± 0.4 V, 12 h operating time, LPF 100 Hz, BLE, CSV, EDF+ formats, FCC ^21^ and CE certified	[146,148]
BrainBit DragonEEG	21/Dry or wet (Au or Ag/AgCl)	500 Hz	EMG/ECG/ EOG	Research, Neuro-technology, Neurofeedback	Fp1, Fpz, Fp2, F7, F3, Fz, F4, F8, T3, C3, Cz, C4, T4, T5, P3, Pz, P4, T6, O1, Oz and O2 electrodes, 3 cap sizes, Input range ± 0.4 V, BLE, Open SDK, 8 h operating time, FCC, CE certified	[147]
Neuro-electrics Enobio	8, 20, 32/Wet or dry	500 Hz, 24 bit (0.05 µV)	ACC	Clinical research BCI, Neurofeedback	LPF 125 Hz, 6 cap sizes, Noise 1 µV RMS, Input impedance 1 GΩ, CMRR ^22^ −115 dB, Wi-Fi, MicroSD ^23^ card, 5 h (24 h) operating time, Dimensions 89.1 × 61.1 × 23.8 mm, Weight 97 g, CE certified	[149]
Neuro-electrics Startsim	8, 20, 32/Wet	500 Hz, 24 bit (0.05 µV)	ACC, Stimulation	Research, Clinical use	BPF 0–125 Hz, Noise 1 µV RMS, Input impedance 1 GΩ, CMRR −115 dB, Wi-Fi, MicroSD card, Li-Ion battery, 4 h operating time, Dimensions 163 mm × 79 mm × 70 mm, Weight 226 g, FDA not clinical, CE	[150]

^1^ Electroencephalography. ^2^ Electrocardiography. ^3^ Electromyography. ^4^ Electrooculography. ^5^ Functional near-infrared spectroscopy. ^6^ Accelerometer. ^7^ Brain–computer interface. ^8^ Root mean square. ^9^ Bluetooth. ^10^ Food and Drug Administration. ^11^ European conformity. ^12^ Inertial measurement unit. ^13^ Band-pass filter. ^14^ Common mode sense and driven right leg. ^15^ Analog-to-digital converter. ^16^ Analog front end. ^17^ Bluetooth low energy. ^18^ Gyroscope. ^19^ Low-pass filter. ^20^ Software development kit. ^21^ Federal communications commission. ^22^ Common-mode rejection ratio. ^23^ Micro secure digital.

### 2.2. Smart EEG Patches and Tattoos

**Figure 2:**
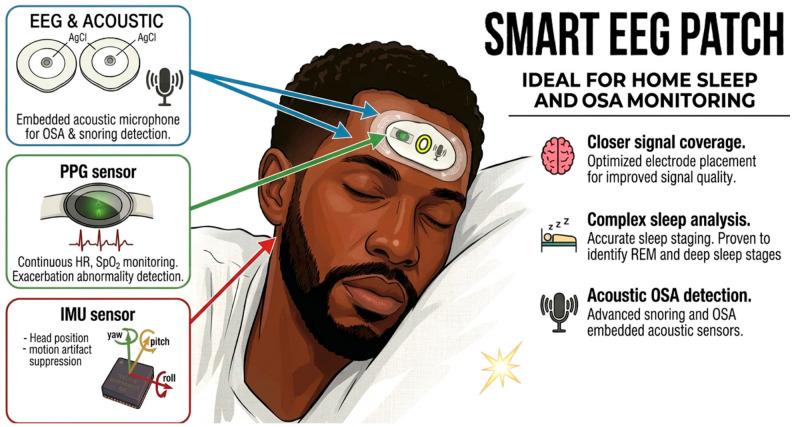
Smart EEG patch.

Unlike traditional designs, patch-like and tattoo-like EEG systems (Figure 2, Table 4) use minimalist, ultra-thin electronics that adapt to the skin, making them highly promising for future monitoring. They record EEG waves localized mainly on the forehead or temples. Due to their small size and lightweight nature, they do not interfere with the wearer, offering minimal restrictions, comfort, and discretion, making them suitable for long-term monitoring [155]. The primary disadvantage is their limited spatial coverage, which prevents recording from a larger scalp area. However, for specific daytime and sleep applications, the minimalism of these devices is sufficient, as the resulting signals retain enough information to determine sleep stages, detect drowsiness, or perform spectral analysis, especially when combined with appropriate signal processing and additional sensors [156,157].

To study sleep stages and sleep-related events, these systems use additional sensors to measure movement, heart rate, and autonomic signals, facilitating automated evaluation in home environments. Thanks to this multimodal integration, these systems can also monitor neurophysiology, fatigue, alertness, and cognitive status during daily activities. This data fusion is not only an improvement but also compensates for the reduced number of EEG channels, ultimately enhancing the interpretability and informativeness of the measured data [158]. However, generalizing this data for clinical sleep diagnostics remains an unresolved issue. For this reason, EEG patches and tattoos are not considered a replacement for head-worn EEG but rather complementary technologies. As their development progresses, their reach may gradually expand into applications where head-worn devices seem impractical. While many of these systems remain experimental, they are increasingly being implemented in practice, offering promising future potential due to the diverse materials that can be utilized [155].

Smart EEG patches are adhesive sensory units, sometimes paired with small auxiliary devices, constructed from ultra-thin, flexible materials that adhere closely to the skin. They usually contain one to two miniaturized electrodes, with optional integration of EOG, EMG, SpO_2_, PPG sensors, and a skin temperature sensor. Electrode materials include thin-film metals (such as gold or silver), conductive polymers, graphene-based conductors, and hybrid nanocomposites, often embedded in elastomeric carriers for better skin adaptability [155,158]. Patches offer easy application outside the hair area, comfort, and stable skin contact achieved through either medical adhesives or dry adhesion based on the van der Waals method. The study of adhesive materials remains an active field due to challenges such as skin irritation, sweat accumulation under the patch during prolonged wear, and signal degradation over time. These devices find applications in motion tracking, home sleep monitoring, circadian rhythm studies, seizure and epilepsy detection, and emotion recognition. Due to their miniaturized form factor, they are also suitable for neonatal and pediatric EEG screening [159,160,161].

Commercially available devices in this category include X-trodes with EmotivPRO platform. These wireless, non-invasive adhesive electrodes conform closely to the skin, without gel or saline need, and provide up to 10 h of continuous recording. The need for disposable electrodes, however, increases operational costs. Their unobtrusive design allows integration into virtually any activity, facilitating data acquisition in neuroscience, sleep studies, cognitive-state and human-performance research, and mobility research [162,163].

The Cognionics (CGX) Flex EEG patch is a lightweight, rapid-deployment device designed for reliable data collection in home and ambulatory environments. It features disposable electrodes, and data can be stored locally on an SD card or streamed in real time via Bluetooth to the CGX Flowpoint software. By utilizing advanced amplification techniques, it effectively suppresses ambient electrical noise. This patch is suitable for research purposes, such as sleep monitoring, sleep stage-classification, ERP studies, cognitive neuroscience, and mobile EEG research. Validation studies have demonstrated reliable detection of N200 and P300 event-related brain potentials [164,165,166].

Neurosteer is a patch-based EEG system that utilizes external electronics rather than a fully integrated patch form factor. It comprises three electrodes built into a conductive disposable adhesive strip applied to the forehead, which can record EEG for 12 h. The device has been approved for medical use and has been applied in clinical research, clinical trials, early detection of neurodegenerative diseases, assessment of brain maturity in premature babies, neurofeedback, and the quantification of specific brain metrics [167,168].

The UMindSleep sleep tracker is a compact single-channel EEG patch that combines sleep monitoring with HR, SpO_2_, snoring, body position and jaw temperature. Paired with the UMindSleep app, it offers sleep analysis and suggestions for improvement based on the American Academy of Sleep Medicine (AASM) and clinical criteria. AI algorithms are employed to achieve the clinical standard of professional monitoring. The device is intended for individuals seeking to improve their sleep quality, with a recommended monitoring frequency of at least three times a week [169,170].

UMindMirror is the world’s smallest EEG system designed to provide neurofeedback, sleep monitoring with automated insights for sleep hygiene, meditation tracking, concentration training, and health monitoring. It features a prolonged battery life of up to 70 h and offers an SDK for developing custom applications [171,172].

Epilog is a two-electrode EEG patch developed for long-term seizure activity monitoring. Its lightweight and water-resistant design makes it particularly suitable for pediatric patients from 1 year of age. Monitoring periods are usually 7 days, after which the patch is removed and returned to the clinician, eliminating the need for manual seizure diaries by automatically recording both the frequency and duration of events. The patch is still in the research stage and is not yet commercially available [173,174,175].

Tattoo-like EEG systems extend the category of patches utilizing ultra-flexible materials, such as conductive graphite-based materials or printed polymer inks, that conform to the skin to mimic temporary tattoos. They function as dry electrodes characterized by mechanical compliance, minimal artifacts, elasticity, comfort, and extreme thinness. These systems hold promises for long-term EEG measurements outside the laboratory, sleep monitoring, and pediatric or neonatal applications due to their small footprint. However, they suffer from disadvantages, such as fragility due to their thinness and limited lifespan [155]. Experimental prototypes demonstrate the feasibility of measuring selected sleep functions, with forehead or ear electrodes achieving performance comparable to conventional electrodes [157]. Most tattoo-like EEG systems remain in the research phase or exist only as early prototypes; while the tattoo electrodes themselves are elegantly designed, the main limitation lies in the bulky external electronics and data transmission modules, preventing commercial availability. Nevertheless, emerging evidence indicates that tattoos are not only convenient but also comparable in performance to commonly available devices [176].

Currently, while the tattoo electrodes reside directly on the skin, the overall system still requires an amplifier, battery, and wireless module. Consequently, an ultra-thin wire leads from the tattoo electrodes to a small module that can be glued behind the ear, on the neck, or on the forehead, ensuring minimal behavioral interference [177,178]. Ferrari et al. showed that tattoo electrodes made of conductive polymer printed by inkjet printers are powerful, compatible with magnetoencephalography, and suitable not only for EEG but also for other electrophysiological signals measured on the skin. Their exceptionally close contact with the skin allows for high-quality recordings over time [178].

Direct printing of EEG e-tattoos onto the scalp offers a safe, personalized, and hair-compatible layout. These e-tattoos are self-drying, do not require additional conductive gels, and adhere tightly to the skin. Vasconcelos et al. have shown that such printed sensors are comfortable, long-lasting, and provide high-quality signal recording. They printed the electrodes using individual 3D head scans to design the sensor layout and used a 5-axis microjet printing robot to create precise and personalized EEG electronic tattoos across the entire scalp [177]. Huh et al. used such electrodes to measure mental load. These sensors, consisting of a polyurethane (APC-GPU) matrix with a composite coating of poly(3,4-ethylenedioxythiophene) and graphite, were adhesively attached to the forehead and recorded EOG in addition to EEG [179]. Similarly, Shustak et al. demonstrated the ability to monitor sleep and its stages, simultaneously capturing EEG, EOG and EMG signals [180].

**Table 4:** Smart EEG patches.

Device	EEG ^1^ Channels/Electrode Type	Sampling Rate/Resolution	Other Sensors	Applications	Other Specification	Ref.
X-trodes EmotivPRO	2/12/16 Dry	250 Hz, 16 bit (0.763 μV)	EOG ^2^, EMG ^3^ IMU ^4^	Research, sleep monitoring	BPF ^5^ 0.32–750 Hz, Input range ± 25 mV p-p, Noise 2μV RMS ^6^, Input impedance 10 MΩ, BT ^7^, Dimensions 48 × 38 × 19 mm, Weight 28 g, 8 h operating time, FDA ^8^	[163]
CGX EEG Patch	2/Dry/Foam + Hydrogel	500 Hz, 24-bit	ACC ^9^	Home EEG	BPF 0–131 Hz, Input range ± 300 mV, CMRR ^10^ 90 dB, Input impedance 100 MΩ, Noise 0.6 µV RMS, BLE ^11^, SD ^12^ card, Raw data, EDF or CSV format, Weight 40 g	[164]
Neurosteer	3/Dry	500 Hz	-	Research, Clinical trials	Fp1, Fp2, Fpz electrode, BT, Weight 55 g, 10 h operating time, FDA Class II	[168]
UmindSleep	3/Dry	256 Hz, 16 bit	PPG ^13^, ACC, GYRO ^14^, TEMP ^15^, Microphone, Pressure	Sleep tracking	AI ^16^ evaluation and disorder diagnosis, snoring, BT, Li-ion battery, Weight 15 g, CE ^17^	[169]
Umind Mirror	4/Dry	256 Hz 16 bit	ECG ^18^, ACC, GYRO	BCI ^19^, Sleep management, meditation	Input noise 4 μV, CMRR 129 dB, Snore data, Open SDK ^20^, BT, Li-Po battery, 10–12 h operating time, Weight 5.2 g, FDA, CE	[171,181]
Epilog	2/Wet (Au)	512 Hz, 10 bit	-	Epilepsy monitoring	Adhesive electrodes with hydrogel, hydrocolloids, BT, EDF data, 7d operating time, FDA	[175,182]

^1^ Electroencephalography. ^2^ Electrooculography. ^3^ Electromyography. ^4^ Inertial measurement unit. ^5^ Band-pass filter. ^6^ Root mean square. ^7^ Bluetooth. ^8^ Food and Drug Administration. ^9^ Accelerometer. ^10^ Common-mode rejection ratio. ^11^ Bluetooth low energy. ^12^ Secure digital. ^13^ Photoplethysmography. ^14^ Gyroscope. ^15^ Temperature. ^16^ Artificial intelligence. ^17^ European conformity marking. ^18^ Electrocardiography. ^19^ Brain–computer interface. ^20^ Software development kit.

### 2.3. In-Ear and Headphone-Based EEG Devices

**Figure 3:**
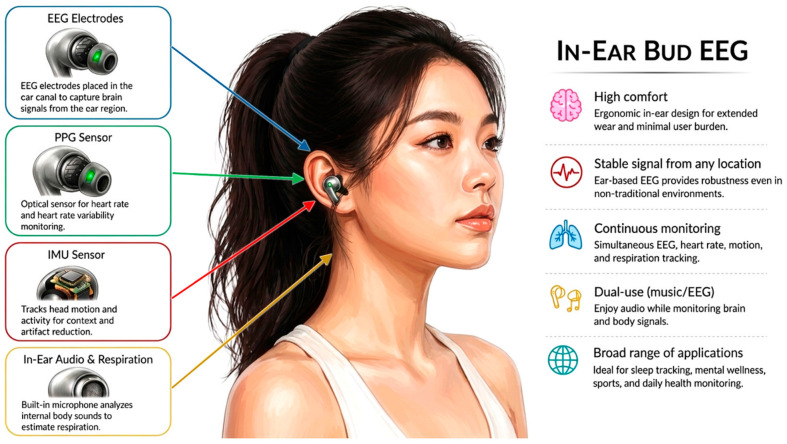
Earbuds with EEG.

This category includes devices with dry or semi-dry electrodes placed inside or around the ear canal, enabling EEG recording with relatively stable signal acquisition during daily activities (Figure 3, Table 5). Their application around the ear suggests the possibility of integration into hearing aids to ensure high wearability, discretion, comfort, and stable placement of electrodes that are relatively resistant to head movement. The earpieces used can be generic or even custom-shaped to precisely match the geometry of the ear canal, utilizing comfortable silicone or elastomer materials for better adaptation to the wearer [156,183]. They are well accepted by users and find applications in the monitoring of sleep, sleep apnea, wakefulness, epileptic seizures, cognitive load, and BCI, as well as in passive long-term monitoring. Other sensors such as microphones for snoring and breathing detection, motion sensors, or PPG for HR tracking suitably complement the cutaneous EEG signal [184].

The limitations are like those of patches and tattoos, namely that they cover only a small part of the head with a small number of electrodes, thereby failing to capture full-scalp dynamics. However, they rely on mechanical pressure rather than adhesives, which significantly reduces the possibility of skin irritation [156,183]. The variable anatomy of the ear represents a significant challenge, as devices frequently require individual customization. Furthermore, sleeping on the side with these devices may cause discomfort, and sensitivity to jaw movement can deform the ear canal, leading to transient artifacts.

Although ear EEG captures lower signal amplitudes than traditional scalp EEG, it has been shown to be sufficient for monitoring sleep-related rhythms, such as sleep spindles, alpha activity, and slow waves. An additional advantage of electrodes around the ear is that they eliminate the need to wash conductive gels or solutions from the hair. While many of these systems remain in the research phase, their strong correlation with conventional scalp EEG underlines their substantial potential for ambulatory neurophysiology and sleep monitoring [156,184,185,186].

In addition to headsets, OpenBCI offers an unobtrusive EEG measurement solution around the ears. The cEEGrid is a flexible, reusable, and comfortable system for unobtrusive around-the-ear EEG monitoring allowing the recording of other electrophysiological signals such as ECG or EMG, although ECG is considered a derived signal in this configuration [142]. Thanks to the CytonDaisy biosensor board, it can record up to 16 channels. The electrodes are typically Ag/AgCl, but the gold-plated option provides an effective alternative, exhibiting lower impedance in the 1–1000 Hz range. Electrodes are attached using disposable adhesive strips and require electrolyte application before use, making the assembly a bit more time-consuming [186]. Although a supporting structure such as a headband, cap, or VR headset is required, cEEGrids provide a comfortable solution for long-term monitoring. They have been successfully used in sleep-stage classification, mental workload assessment, mobile EEG studies, real-world cognitive monitoring, and bruxism detection [10,185,187,188,189]. Validation study [10] showed reliable recordings for 6–7 h and successful smartphone-based operation outside laboratory environments.

An interesting device, the Emotiv MN8 headphones combine EEG sensing with conventional headphone functionality to monitor cognitive performance, attention, and mental states. The device is discreet and comfortable, can be applied in less than 1 min without gels or adhesives, and incorporates motion sensing to improve signal interpretation. The device functions like regular headphones and functionally allows users to listen to high-quality audio or receive phone calls [190]. Applications include cognitive-state monitoring, neurofeedback, productivity assessment, and human-performance optimization. Emotiv also introduced the latest wireless MW20 headphones with EEG, noise cancelation support, and audio playback in a lightweight, discreet and sweat-resistant design. Using dry brush electrodes positioned in each ear, the system monitors stress, recovery, concentration, and mental wellness, while HRV-based metrics can be used to estimate cognitive age and support personalized performance management [191].

Similarly, the Smartbuds headphones are designed to optimize sleep, focus, or relaxation based on the wearer’s needs through adaptive audio and music to support the brain. The application of advanced machine learning enables the classification of sleep stages with performance comparable to traditional laboratory EEG, highlighting their potential for home sleep monitoring and personalized neurofeedback applications [192].

The MW75 Neuro LT headphones from Neurable integrate electrodes into memory foam ear pads around the ear rather than within the ear canal. The system combines premium sound with real-time monitoring of attention, concentration, and stress, supporting productivity optimization, cognitive-state assessment, and neuroadaptive human–computer interaction [193,194].

The BrainBit headset analyzes EEG for cognitive performance optimization, offering exercises focused on concentration, relaxation, and general neurofeedback. An open SDK further supports research and custom neurotechnology development [195].

The Brainlink Tune headphones utilize dry sensors for EEG recording, neurofeedback, cognitive training and meditation. Dedicated study and work modes are integrated with an AI-based assistant designed to support concentration and cognitive performance [196].

Enophones are EEG-enabled audio devices designed for neurofeedback, mental health tracking, sleep monitoring, and cognitive-performance assessment. By combining EEG measurement with adaptive audio delivery, they provide personalized experiences that respond to the user’s mental state [197].

**Table 5:** In-ear and headphone-based EEG.

Device	EEG ^1^ Channels/Electrode Type	Sampling Rate/Resolution	Other Sensors	Applications	Other Specification	Ref.
OpenBCI cEEGrids	8–10/Wet (Au or Ag/AgCl)	125/250 Hz	EOG ^2^ (adjacent) ECG ^3^ (derived)	Sleep staging, ambulatory EEG, BCI, bruxism detection	Flexible printed electrodes, ADS1299 AFE ^4^, 500 mAh Li-ion battery, 10 h operating time	[185,186]
Emotiv MN8	2/Dry	250 Hz, 14-bit (0.51 µV)	Microphone, ACC ^5^, GYRO ^6^	EEG research, discreet attention research	Conductive elastomer earbuds, BPF ^7^ 0.5–43 Hz, dynamic range 10.4 mV (p-p), Active Noise Cancelation, BLE ^8^, 6 h operating time	[190]
Emotiv MW20	2/Dry Conductive elastomer	128 Hz, 16-bit	Motion	BCI ^9^, games, cognitive metrics	Earbuds, 8 h operating time	[191]
Smartbuds	6/Dry	1000 Hz, 24 bit	ACC, GYRO	Sleep, focus, relaxation	Slow wave stimulation, 6 mm audio driver, 35 mAh battery, 9 h operating time, BT ^10^, weight 5 g (one Smartbud)	[192]
Neurable	12/Dry (fabric)	500 Hz	IMU ^11^, Microphone	Neurofeedback, stress management	BPF 0–131 Hz, BT, 40 mm Beryllium audio drivers, 8× Microphone, ANC ^12^, 12 h operating time	[193,194]
BrainBit EEG Headphones	4/Dry (Au)	250 Hz	Microphone	Focus, relaxation training, education	Au-plated spring-loaded electrodes, 2 × 40 mm speaker, BT, Open SDK ^13^, CSV, EDF+ formats, 8 h operating time, CE ^14^ and FCC ^15^ certified	[195,198]
BrainLink TUNE	1/Dry (Au)	512 Hz	GYRO	Neurofeedback, meditation, study	8 brain wave bands, BLE, 220 mAh battery, 8 h operating time, dimensions 16 × 16 × 3 cm, Weight 40 g, FCC, CE certified	[196]
Enophones	4/Dry (Au)	250 Hz	ACC	Neurofeedback	A1, A2, C3, C4 electrodes, noise 0.2 µV RMS ^16^, BT, 14 h operating time, weight 370 g	[197]

^1^ Electroencephalography. ^2^ Electrooculography. ^3^ Electrocardiography. ^4^ Analog front end. ^5^ Accelerometer. ^6^ Gyroscope. ^7^ Band-pass filter. ^8^ Bluetooth low energy. ^9^ Brain–computer interface. ^10^ Bluetooth. ^11^ Inertial measurement unit. ^12^ Active noise cancelation. ^13^ Software development kit. ^14^ European conformity. ^15^ Federal Communications Commission. ^16^ Root mean square.

### 2.4. Glasses-Integrated EEG Devices

**Figure 4:**
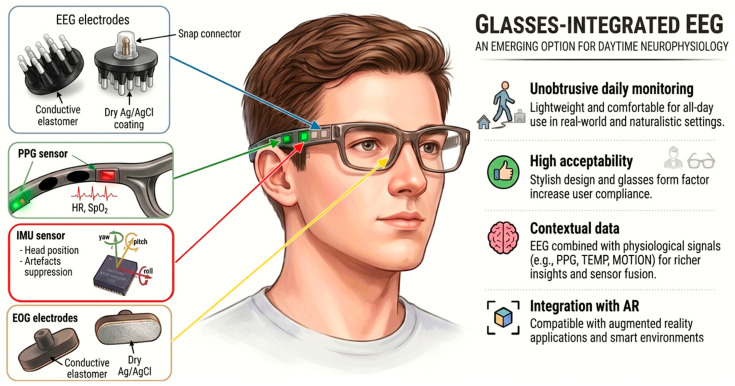
EEG smart glasses.

EEG glasses (Figure 4, Table 6) represent a highly practical tool for monitoring in real-world environments. They have a specific shape that limits data acquisition to the area of the head where the frame extends, integrating conventional dry electrodes around the eyes, on the temples, or on the bridge of the nose to capture frontal and periorbital EEG signals. Since many people wear glasses daily, these sensors can be integrated without significant restrictions on the wearer. Furthermore, wearing them is easy and does not attract unwanted attention [199,200]. They can continuously monitor neurophysiology, including sleep, although they may be less convenient compared to previous groups of devices. EEG glasses are unlikely to replace other systems in sleep measurement; however, they are highly suitable for measuring neurophysiology during the day, monitoring attention, and integrating with AR systems or HMIs due to their easy use in everyday life [199,200]. Also, the incorporation of additional sensors brings a more holistic assessment of human physiology. For example, EOG recording is valuable for distinguishing neural activity from artifacts arising from muscle activity and eye movements, while IMU sensors are utilized for artifact detection and noise removal. These and other physiological sensors provide multimodality, enabling a more comprehensive view of the individual’s physiology [199,200]. Movement artifacts can be a problem, since glasses are typically not tightly secured to the face and exhibit more relative motion during physical activity than other EEG systems where the electrodes fit tightly. This often results in lower signal quality, but multimodal sensing and advanced signal processing significantly improve the acquisition of valuable data [200]. In the literature, devices such as Attentive, e-Glass, and GAPses are highlighted as very promising for BCI and everyday neurophysiological applications.

AttentivU smart glasses integrate EEG, EOG and fNIRS sensors together with additional sensors to improve the measurements accuracy. Currently in early commercialization, they are primarily utilized in research on BCI, cognitive workload, fatigue, stress monitoring, attention assessment, and productivity optimization. EEG electrodes are positioned behind the ears, EOG sensors on the nose bridge, and fNIRS modules on the sides above the ears [201,202,203].

The e-Glass system enables real-time monitoring of epileptic seizures and cognitive states. Soft, dry EEG electrodes and embedded electronics are fully integrated into the frame, eliminating external cables and demonstrating an unobtrusive and elegant solution. However, their use increases sensitivity to noise due to the impedance mismatch between the skin and the measurement circuit. This problem is addressed by using active electrodes with voltage followers directly at the electrode site, which increases the input impedance, minimizes signal loss, and enables local signal amplification. The system has been investigated for seizure detection, cognitive monitoring, and unobtrusive ambulatory EEG applications [204].

GAPses smart glasses combine EEG and EOG recording using dry soft electrodes and an integrated, ultra-low power RISC-V processor. With a battery life that extends beyond 22 h, they are suitable for long-term monitoring. Applications include HMI, biometric identification, cognitive-state assessment, and real-time EEG/EOG analysis. Integrated rigid-flex printed circuit boards and configurable EEG/EOG acquisition modes help reduce noise and motion artifacts. A separate board allows channel selection and switching between EEG-only and combined EEG/EOG configurations [205].

AAVAA are glasses designed primarily for BCI and hands-free device control. Using dry, highly conductive electrodes, they provide more than 20 h of recording and are optimized for low-noise acquisition with minimal electrode-polarization impedance effects. Data can be stored locally on the device or in the cloud, supporting AI-based classification of cognitive and neural states [206].

The Neuropilot smart glasses are designed for attention and concentration monitoring alerting the wearer when distraction is detected. An integrated AI detects distraction-related brain patterns and provides real-time feedback to support cognitive performance. The device can be fitted with prescription lenses, allowing a solution for eyeglass wearers who can leverage their everyday eyewear without requiring additional EEG hardware while simultaneously monitoring cognitive state [207].

**Table 6:** Glasses-integrated EEG.

Device	EEG ^1^ Channels/Electrode Type	Sampling Rate/Resolution	Other Sensors	Applications	Other Specification	Ref.
AttentivU	3/Dry (Ag/AgCl)	1000 Hz, 10 bit	Optional EOG ^2^, fNIRS ^3^, Microphone	BCI ^4^, Research, Neurofeedback	Processing power 192 mW, 7.5 h operating time, BLE ^5^, Wi-Fi, Dimension 16.5 × 14.6 × 6.4 cm, Weight 57 g	[201]
e-Glass	4/Dry	250 Hz, 24 bit	ACC ^6^	Research, Epilepsy monitoring	F7, F8, T7, T8 electrodes, HPF ^7^ 30 Hz, ADS1299 AFE ^8^, CMRR ^9^ 102 dB, Li-ion battery, 12 h operating time	[204]
GAPses smart glasses	8/Dry (Brush pins)	500 Hz, 24-bit	EOG ACC	Research, Cognitive load, Biometric, BCI	TP9, TP10 electrodes, BPF ^10^ 0.5–100 Hz, Notch filter, RISC-V ^11^ processor GAP, Consumption 16.28 mW, BT ^12^, 75 mAh battery, 12 h operating time, Dimension 160 × 145 mm, Weight 40 g	[205]
AAVAA	4/Dry	125 Hz, 24 bit	EOG IMU ^13^	BCI	Control nearby devices, Data transfer 2 Mbps, Ultra-low consumption, BT, 20 h operating time, CE ^14^	[206]
Neuropilot	4/Dry	256 Hz	HR ^15^ and breathing	Neurofeedback	Noise 5 µV RMS ^16^, 5 h operating time, Weight 45 g	[207]

^1^ Electroencephalography. ^2^ Electrooculography. ^3^ Functional near-infrared spectroscopy. ^4^ Brain–computer interface. ^5^ Bluetooth low energy. ^6^ Accelerometer. ^7^ High-pass filter. ^8^ Analog front end. ^9^ Common-mode rejection ratio. ^10^ Band-pass filter. ^11^ Reduced instruction set computer-v. ^12^ Bluetooth. ^13^ Inertial measurement unit. ^14^ European conformity. ^15^ Heart rate. ^16^ Root mean square.

## 3. Signal Quality and Data Processing Algorithms

Signal quality and data processing play an important role in accurate identification and validation of captured data. This section focuses on how each device category manages signal quality and processes EEG and additional sensor data, especially in automated or real-time settings.

### 3.1. Signal Acquisition and Quality Metrics

Smart wearable EEG devices differ mainly in the number of channels, the electrode–skin interface, and the recording place. These factors strongly affect the quality of the recorded EEG. In practice, wearable systems range from very low-channel designs, such as single-channel forehead or ear-centered devices, to higher-density wearable caps with 16, 24, 32, or more channels. However, most consumer headbands and many compact wearable systems use only a few channels and are usually focused on the frontal or frontotemporal regions of the head [1,11,18]. The need for a high number of channels is also application-dependent, with research showing that devices with only a pair of electrodes can also provide recordings suitable for targeted applications [10,156,208,209]. A standardized 10–20 arrangement is commonly used as a reference [210], while many wearable devices do not use the full 10–20 system. The most common headsets use frontal, temporal, and parietal regions (F3, F4, T7, T8, Pz, AF3, AF4), headbands commonly use frontal positions (Fp1/2, AF7/8, Fpz), caps often use the full 10–20 system [1,6,103,205,208], patches are usually placed frontally (Fp1/2), tattoo-like systems allow flexible placement but are often located on the frontal region, in ear EEG is associated mainly with temporal or around-ear locations (Tp9/10), and glasses-based system use frontal and temporal regions. Overall, frontal and temporal regions are frequently used for wearable EEG electrode placement. This region allows easier electrode contact because it is less affected by hair. As devices become smaller, electrode placement tends to shift toward the frontal area [1,6,11,103,205,208].

This limited channel count is an important trade-off. Fewer electrodes make the device easier to wear, faster to set up, and more comfortable for home or long-term use, but they also reduce spatial coverage and signal redundancy. As a result, low-channel systems are more sensitive to local signal loss, poor contact, and motion-related contamination. In contrast, devices with more channels can capture a wider range of brain activity and allow better identification of corrupted signals, but they are usually more complex and less comfortable for everyday use [1,11].

Across wearable EEG categories, reported sampling rates and signal resolutions vary widely, and consumer-facing technical documentation is often incomplete. For this reason, performance is often judged not only based on datasheets, but also from validation studies and systematic reviews. The sampling rate for EEG recording depends on the frequency composition of the signal and the target application. Most devices converge around 250 Hz, while 100 Hz would be sufficient for EEG. Applications such as neurofeedback and sleep monitoring typically operate in the range of 128–256 Hz, while BCI and ERP studies may require higher sampling rates of up to 500 Hz or more, although 256 Hz is also used, as shown in Table 1 [211,212,213,214,215]. The most reported signal-quality indicators include electrode–skin impedance, noise level or spectral floor, wireless dropouts or packet loss, and signal-to-noise ratio (SNR) in relevant frequency bands such as alpha activity or sleep spindles [6,11]. In sleep-related applications, additional practical indicators are also important, such as signal stability across the whole night and the ability to preserve usable data during body movement or changes in posture.

The quality of wearable EEG does not depend only on hardware parameters. It is also shaped by the recording location. Forehead and frontotemporal systems are easier to place and often provide stable contact, but they are more exposed to eye-movement and facial-muscle artifacts. Ear-centered systems usually provide fewer channels and lower amplitudes, but they may benefit from relatively stable mechanical placement during long recordings. Patch-based systems can achieve good skin contact, but only over a small recording area. Because of these differences, wearable EEG quality should always be interpreted in relation to the intended application, rather than only according to a single technical metric [1,11,18].

Overall, reviews suggest that wearable EEG can reach moderate-to-substantial agreement with PSG and other reference systems, but performance depends strongly on device design, channel count, electrode contact, and recording conditions [6,11]. This is especially important for low-channel devices, where strict signal-quality control is needed because there is little redundancy for detecting and rejecting corrupted segments [1].

### 3.2. Electrode Interface: Dry vs. Semi-Dry Performance in Long-Term Use

Most smart wearable EEG devices use dry or semi-dry electrodes because they are faster to apply and more practical than traditional gel-based electrodes. This is important for home monitoring, repeated use, and long recordings during sleep or daily activities. However, easier use usually comes with a trade-off in signal stability and contact quality [11,18].

Dry electrodes do not require gel, which makes them more comfortable and easier to use in real-world settings. At the same time, they often have higher and more variable electrode–skin impedance than wet electrodes. Because of this, dry systems are usually more sensitive to motion, pressure changes, and unstable contact with the skin. These effects can increase noise and make the signal less stable, especially during long recordings. Modern designs try to reduce these problems by using improved materials and structures, such as conductive polymers, spring-loaded contacts, soft electrode surfaces, flexible conductive composites, or micro-texturized ultra-soft dry electrodes designed to improve skin adhesion and reduce motion-related artifacts. The long-term performance of dry electrodes also depends on the electrode material and surface design, because these factors influence impedance, skin conformity, comfort, and motion sensitivity [19,20,21,216,217,218].

Semi-dry electrodes try to solve part of this problem by using a small amount of electrolyte. This usually improves electrical contact and can reduce impedance compared with fully dry systems. As a result, semi-dry electrodes may provide more stable signals, especially at the start of recording. However, this advantage may decrease over time. During long-term use, the small amount of electrolytes can dry out, contact quality can change, and skin irritation may appear, especially in overnight monitoring or in conditions with sweating and pressure from the device [11,216].

In-ear systems have a somewhat different situation. Their performance depends less on forehead or scalp contact and more on how well the earpiece fits the ear canal or the area around the ear. A well-fitted ear electrode can be mechanically stable and may work well during long recordings. On the other hand, ear anatomy differs strongly between users, so comfort and coupling quality are not always the same. Poor fit can reduce signal quality, and long-term wear may become uncomfortable, especially during sleep or side sleeping [18,208,216]. Glasses-integrated EEG systems present a related challenge: they also commonly use dry electrodes, but because the frame is less tightly coupled to the head than a headband or patch, small shifts during movement may reduce contact stability and increase motion-related noise [205,219].

Overall, long-term wearable EEG recording is affected not only by the type of electrode, but also by gradual changes in electrode–skin coupling over time. These changes may appear as impedance drift, baseline instability, or slowly changing noise characteristics. For this reason, the choice between dry and semi-dry electrodes is not only a question of comfort, but also of how stable and reliable the signal remains during the full recording period [11,216].

### 3.3. Artifact Landscape and Artifact Reduction Methods

Wearable EEG is strongly affected by artifacts, and the type of artifact usually depends on where the electrodes are placed and how the device is attached to the body. In other words, artifact contamination in wearable EEG is not random. It follows the mechanical and anatomical conditions of each device form factor. For this reason, artifact interpretation is an important part of signal evaluation in wearable systems [219].

In headband-based systems, which are usually placed on frontal or frontotemporal areas, the most common artifacts come from eye blinks, eye movements, facial muscle activity, and head motion. Because these electrodes are close to the eyes and forehead muscles, EOG and EMG contamination is often strong. This is especially important in daily-life recordings, where the user is speaking, moving, or changing facial expression [219,220].

Patch-based systems, especially those placed on the forehead or temple, may also be strongly influenced by eye-related artifacts. In addition, because they rely on adhesive contact with the skin, small shifts of the patch or changes in skin condition may create low-frequency drift or unstable baseline behavior. These effects may become more visible during long recordings, sweating, or repeated changes in posture [216].

In-ear systems are affected by a different artifact profile. Common problems include jaw-movement and chewing-related EMG, movement of the earpieces, and mechanical deformation around the ear during speaking or head movement. At the same time, ear-centered systems may benefit from relatively stable placement during sleep or quiet activities. This means that the same form factor may be advantageous in one situation and more difficult in another, depending on user behavior and recording conditions [18,208].

Several signal-processing approaches are commonly used to reduce or mark artifacts. Basic digital filtering is usually applied as an early preprocessing step to attenuate slow drift, high-frequency noise, and mains interference; however, filtering alone cannot reliably remove structured EOG, EMG, or motion-related artifacts. More advanced methods include independent component analysis (ICA), which is widely used to remove ocular and muscle-related components in research-grade processing [221,222]. Another important approach is artifact subspace reconstruction (ASR) and related methods, which are useful for reducing short, high-amplitude disturbances in mobile EEG. However, these methods are sensitive to parameter selection and should be applied carefully, because overly aggressive cleaning may also distort useful neural information [149,150,155].

Overall, artifact reduction in wearable EEG is not only a technical preprocessing step. It is closely linked to device design, recording location, and the intended application. A method that works well for a multi-channel research headset may not be equally suitable for a low-channel forehead device or an in-ear system. For this reason, artifact handling in wearable EEG should be chosen with respect to both the signal properties and the practical conditions of measurement [219,220].

### 3.4. Typical Processing Pipeline

A typical wearable EEG processing pipeline usually starts with signal-quality control. This is especially important in wearable systems because they often use only a small number of channels, so there is less redundancy than in full PSG or laboratory EEG. If one channel is strongly corrupted, a large part of the recording may become difficult to use. For this reason, early detection of poor signal quality is an important first step [1,220].

The first stage usually includes basic signal checks. These checks may identify missing samples, signal saturation, unstable baseline, excessive drift, or poor electrode contact. In wearable recordings, such problems may appear because of body movement, changing pressure on the device, loose contact, or wireless transmission issues. Detecting these problems early helps prevent later analysis from being based on low-quality data [219,220].

After signal checking, standard preprocessing is usually applied. This often includes resampling, if needed, followed by digital filtering. In sleep-oriented EEG pipelines, reported filter settings often use a lower cutoff around 0.1–0.5 Hz and an upper cutoff around 35–45 Hz; a common sleep-staging-oriented example is approximately 0.3–35 Hz. Other wearable sleep EEG configurations may retain a wider band, for example, 0.1–100 Hz in ear-EEG studies, together with notch filtering at the local mains frequency, typically 50 or 60 Hz, and in some cases also at harmonic frequencies such as 100 Hz to suppress power-line noise. In more general wearable EEG, cognitive-state, or BCI applications, passbands such as 0.5–40 Hz, 0.5–45 Hz, or 1–40 Hz may be used, depending on whether slow drift, ocular activity, muscle activity, or higher-frequency EEG components are relevant for the analysis. Several wearable systems also report device-specific acquisition or built-in bandwidths, such as approximately 0.16–43 Hz, 0.5–43 Hz, 0.03–35 Hz, 0.32–750 Hz, or 0.5–100 Hz, showing that practical filter settings vary across hardware platforms and applications. If the device uses more than one EEG channel, re-referencing may also be performed. The exact preprocessing steps depend on the device, the sampling rate, and the final application, but the main goal is the same: to prepare a cleaner and more stable signal for later analysis [24,35,103,163,191,205,208,220,223].

The next stage is artifact handling. In some systems, simple rule-based methods are used to mark segments with strong movement or abnormal amplitudes. In more advanced pipelines, methods such as ICA or ASR may be applied when the signal configuration allows it. In low-channel wearable systems, complex artifact correction is often more limited, so highly corrupted segments may simply be rejected instead of heavily corrected [221,224].

Once the signal is cleaned, the data is usually divided into smaller time segments for analysis. In sleep staging, this is commonly done using fixed-length epochs. These segments can then be processed in two main ways. In traditional pipelines, features are extracted, such as spectral power, band ratios, or simple statistical measures. In more recent approaches, the segmented signal is used directly as input to deep-learning models, either in raw form or after time–frequency transformation [220,223].

Many wearable systems also use multimodal fusion. Signals from accelerometers, gyroscopes, or PPG sensors can help detect motion, body position, pulse-related changes, or possible arousals. These additional signals do not replace EEG, but they can improve the interpretation of difficult segments and make the overall system more robust in real-world conditions [11,219].

Overall, the wearable EEG processing pipeline is not only a technical sequence of steps. It is a practical strategy for working with limited, noisy, and often heterogeneous data. Because wearable devices differ in channel count, sensor combination, and intended use, no single pipeline fits all systems equally well. Still, careful quality control, basic preprocessing, suitable artifact handling, and well-chosen segmentation remain the core elements of most wearable EEG workflows [1,220].

### 3.5. Embedded or Companion Algorithms

Embedded or companion algorithms play an important role in smart wearable EEG systems. In many devices, the recorded signal is not only stored but also processed automatically to produce a direct output for the user or clinician. Depending on the device, this process may run partly on the device itself, in a connected mobile application, or in external software. The complexity of these algorithms depends on the number of channels, signal quality, available sensors, and the intended application [220].

Sleep staging is currently the most mature application area for wearable EEG. In recent years, many systems have moved from traditional hand-crafted features toward machine learning and deep learning models. These include convolutional neural networks, recurrent models, and more recent transformer-based approaches. Well-known examples such as DeepSleepNet and TinySleepNet show how even limited EEG input can be used for automated sleep scoring when the processing pipeline is designed carefully [220,223,225,226]. In practice, sleep staging is the area where wearable EEG has the clearest pathway from signal acquisition to useful automated output.

Cognitive-state classification, such as mental workload, attention, fatigue, or drowsiness detection, is also widely studied. However, this area is less standardized than sleep staging. Performance depends not only on the signal itself but also on the task design, the quality of the labels, and the differences between subjects. Systematic reviews show that many reported models perform well only in specific experimental settings, while generalization across tasks or users remains more difficult [9,227,228]. For this reason, cognitive-state algorithms in wearable EEG should be interpreted more carefully than sleep-stage classifiers.

Emotion and arousal detection represent another active area of algorithm development. These tasks are often based on valence–arousal models and may use EEG alone or EEG together with other physiological signals. In this case, performance is often strongly influenced by artifacts, subjective labeling, and differences in emotional response between individuals. Some studies suggest that in-ear EEG may offer a practical form factor for more discreet emotion monitoring, but this field is still methodologically heterogeneous [229,230].

Wearable algorithms are also being explored for breathing-related sleep problems, including sleep apnea. In standard PSG, apnea detection usually depends on respiratory sensors, but recent reviews show increasing interest in EEG-based or multimodal detection approaches. In ear-centered and other wearable systems, microphones, motion sensors, or pulse-related signals may be combined with EEG to improve the recognition of breathing disturbances during sleep [208,231]. In such cases, EEG is usually not the only source of information but part of a broader multimodal algorithm.

Many wearable systems now also use neural networks for PPG and motion signals, either together with EEG or in parallel processing streams. These signals can provide information about heart rate, heart-rate variability, breathing-related patterns, movement, and autonomic activation. When combined with EEG, they can improve robustness and help interpret difficult segments, especially when EEG quality is reduced by artifacts or low channel count [11,219,220].

Overall, embedded and companion algorithms are one of the main reasons why wearable EEG has become more useful outside the laboratory. At the same time, algorithmic performance still depends strongly on signal quality, label quality, and the conditions in which the model was developed. Therefore, the value of automated outputs in wearable EEG should always be considered together with the limitations of the underlying signal and the intended use of the system [220,227].

### 3.6. Edge AI vs. Cloud Processing, and Real-Time/Closed-Loop Features

As wearable EEG systems become more advanced, an important question is where the signal should be processed. Some tasks can be done directly on the device or close to it, while others need a more powerful external system. This decision affects speed, battery life, data transfer, and the type of feedback that the system can provide [232,233].

Processing at the edge, meaning on the device itself or on a nearby connected device, is useful for fast and simple operations. These may include basic filtering, signal-quality checks, artifact flags, simple feature extraction, and in some cases lightweight classification. The main advantage of edge processing is low latency. This is important when the system must react quickly, for example, in neurofeedback or other real-time applications. Edge processing may also reduce the amount of raw data that must be transmitted, which can help with privacy, bandwidth, and energy use [232].

Cloud or workstation processing is better suited for more complex tasks. These may include advanced artifact correction, heavier machine learning models, personalization, long-term trend analysis, or report generation. External processing can use more computational power and can support model updates more easily, but it usually depends on stable data transfer and may introduce greater latency. For this reason, it is often less suitable for tasks that require an immediate response [234,235].

In practice, many wearable EEG systems are likely to use a hybrid approach. In such systems, simple and time-sensitive functions are handled at the edge, while more complex analysis is performed later in the cloud or on a workstation. This combination allows the system to balance speed, computational demands, battery use, and data-management needs. It is especially useful in wearable devices, where hardware resources are limited but the application may still require advanced analysis [234,235].

Real-time and closed-loop features are most often discussed in neurofeedback, sleep-related stimulation, and wellness-oriented applications. In these systems, the output of the algorithm is used to guide an immediate response, such as feedback to the user or timed stimulation based on the current brain state. Such approaches are promising, but they are also demanding. Outside the laboratory, reliable closed-loop operation depends strongly on stable signal quality, robust artifact handling, and low-latency processing. Motion, poor contact, and changing environmental conditions can reduce reliability and make real-time decisions more difficult [219,234].

Overall, the choice between edge and cloud processing is not only a technical design issue. It is closely linked to the intended use of the wearable EEG system. Applications that need immediate feedback benefit from local processing, while applications focused on deeper analysis and longitudinal monitoring can benefit more from external computation. Future wearable EEG systems will likely continue moving toward hybrid architectures that combine both approaches in a practical and efficient way [232,233,234].

## 4. Validation and Performance in Targeted Applications

This section summarizes how smart EEG devices have been validated in targeted real-world applications and how their performance compares with clinical reference methods (Table 7). Because these systems differ in electrode interfaces, channel counts, and processing pipelines, reported outcomes are best interpreted in the context of the study design, the reference standard (most often expert-scored PSG), and the chosen metrics (e.g., accuracy, Cohen’s κ, sensitivity/specificity). The following subsections therefore review validation evidence across key use cases—sleep staging, cognitive-state tracking, and selected clinical applications—and conclude with device-type-oriented summaries and compact tables comparing cohorts, sensors, and evaluation protocols.

### 4.1. Sleep Studies: Comparison with Polysomnography

PSG is the gold standard for clinical sleep assessment, combining EEG, EOG, EMG, respiratory measures, and additional physiological signals. Over the last decade, wearable EEG devices have aimed to replicate or complement PSG in home settings while maintaining acceptable accuracy and user comfort [1].

Meta-analysis and systematic reviews show that many wearable EEG systems achieve moderate-to-substantial agreement with standard PSG for sleep-stage classification, although performance varies by device, channel configuration, and the algorithms used. Overall, these systems can reliably distinguish wake, light sleep, deep sleep, and REM sleep, with many studies reporting performance near 80%, although pooled results indicate only moderate-to-substantial agreement overall [6].

Specific validations include the following. The Muse S headband showed strong agreement with PSG for key sleep macro-architecture parameters, particularly total sleep time, sleep efficiency, and wake after sleep onset, and more than 90% of recordings fell within acceptable limits of difference for these metrics [235].

For the Dreem headband (including Dreem 2), multiple studies have confirmed its ability to capture EEG rhythms and classify sleep stages with good agreement with PSG. Metrics such as deep sleep and rapid eye movement (REM) sleep are generally detectable, even if the exact estimates vary across studies [105].

In clinical populations (e.g., Parkinson’s disease), Dreem 2 shows sleep-metric patterns like those observed in healthy participants, with good specificity but a tendency to overestimate certain sleep stages [236].

Additional validation evidence is available for frontopolar wearable sleep EEG systems. In a study of 47 participants who underwent simultaneous PSG and recording with the Sleep Profiler device, automated sleep staging showed substantial agreement with human-scored PSG after expert review, reaching a mean Cohen’s kappa of 0.67 relative to five technologists. In the same publication, a separate clinical cohort of 63 patients was used to examine night-to-night variability and first-night bias across two self-applied home recordings. That analysis showed that several frontopolar sleep biomarkers, including stage-N3-related, spindle-related, and autonomic activation measures, were sufficiently stable for longitudinal assessment. These findings support the use of frontopolar wearable EEG not only for sleep staging but also for repeated biomarker-oriented home monitoring [237]. Recent work using portable BCI-based sleep monitoring systems has further demonstrated that wearable EEG can capture not only macroscopic sleep stages but also microscopic sleep features when compared to PSG, including sleep microarchitecture such as spindles and slow-wave activity, extending the scope of wearable sleep assessment beyond conventional staging approaches [238].

A recent meta-analysis of 43 validation studies reported a pooled sleep-staging accuracy of 78 ± 7% and a mean Cohen’s κ of 0.68 ± 0.10, supporting moderate-to-substantial agreement between wearable EEG systems and PSG. The analysis also showed that performance varies considerably across sleep stages, with the lowest agreement typically observed for N1 sleep and the highest for wakefulness and REM sleep [6]. Overall, wearable EEG appears useful for home sleep monitoring; reviews also indicate that adding EEG provides sleep-stage information that motion-only sensors cannot capture [1,6].

### 4.2. Cognitive State Tracking: Attention, Fatigue, Drowsiness

Wearable EEG systems are also actively evaluated for detecting cognitive states such as attention, fatigue, and drowsiness, which are relevant to neurocognitive research as well as applications in work, transport, and education. In combination with machine learning algorithms, wearable EEG headsets can detect EEG rhythm patterns, such as theta/alpha changes, that are associated with workload, vigilance, and fatigue [228].

Beyond review-level evidence, several device-specific studies show that wearable EEG can detect task-related brain states, although the evidence is more heterogeneous than in sleep research. Validation work with the Emotiv EPOC showed that late auditory event-related potentials were comparable in morphology to those measured with a laboratory EEG system, supporting the use of wearable EEG in attention-related paradigms [29]. More recently, wireless dry-electrode ear-EEG recorded 35 h of electrophysiological data across nine subjects performing drowsiness-inducing tasks and achieved classification accuracies of 93.2–93.3%, demonstrating the feasibility of highly wearable neural monitoring for vigilance detection [209]. Small-sample studies using devices such as Emotiv EPOC X also support the feasibility of multiclass motor-imagery decoding, but they also highlight a persistent limitation of the field: many studies remain task-specific and are based on relatively small cohorts [25].

Such methods can be used for real-time driver-state monitoring or adaptive work environments, although performance depends strongly on electrode design, signal quality, the task protocol, and the labeling strategy [227]. While wearable EEG can distinguish between several mental states, generalizability remains limited because many studies use different paradigms, devices, preprocessing choices, and evaluation metrics [227,228]. For this reason, cognitive-state tracking with wearable EEG is promising, but still less standardized and less directly comparable than sleep staging.

### 4.3. Clinical Potential: Epilepsy, Neurodegeneration, and Cognitive Decline

Wearable EEG systems also show growing potential for clinical applications beyond sleep monitoring. In epilepsy, systematic reviews suggest that wearable EEG technologies can support long-term seizure monitoring, although detecting non-convulsive seizures remains challenging and multimodal sensing may be needed to improve sensitivity [239,240]. Recent work has also demonstrated the feasibility of automated sleep staging in patients with epilepsy using deep learning approaches applied to both conventional EEG and wearable recordings, suggesting that wearable EEG may support sleep assessment in neurological populations where sleep disturbances are common [6].

In neurodegenerative disorders, multimodal wearable EEG combined with AI-based analysis of sleep physiology shows potential for Alzheimer’s disease screening and for broader assessment of cognitive decline. For example, a recent study reported that wearable recordings combining single-channel EEG and accelerometry achieved an accuracy of 0.90 and an area under the curve (AUC) of 0.90 for Alzheimer’s disease detection, suggesting that these physiological features may capture clinically relevant differences beyond hypnogram-level summaries alone. Wearable EEG may also support home-based interventional paradigms. In a pilot study in mild-to-moderate Alzheimer’s disease, 11 adults used the Dreem 2 headband for baseline nights and then for 14 consecutive stimulation nights at home; the study showed that phase-locked acoustic stimulation was feasible and was associated with increased slow-wave sleep [241]. Wearable EEG is also being explored as a tool for monitoring mild cognitive impairment (MCI), where published findings show substantial variability in classification performance but overall support the potential for earlier detection of cognitive deficits in community settings [242]. Taken together, these clinical applications remain largely research-oriented, but they indicate that wearable EEG may provide continuous biomarkers relevant to neurological and psychiatric conditions outside traditional short laboratory recordings.

### 4.4. Published Validation Studies per Device Type

The literature indicates a growing number of wearable-EEG validation studies, with sleep-oriented headbands and frontopolar systems most frequently evaluated against PSG. A meta-analysis identified 43 independent studies comparing wearable EEG with standard PSG for sleep-stage classification, with substantial variation in methodology and performance across individual sleep stages [6]. The meta-analysis further demonstrated that differences in electrode placement, channel count, algorithmic processing, and scoring methodology contribute substantially to inter-study variability, highlighting the need for standardized validation protocols and reporting frameworks [6]. Independent device-level studies now include not only headbands such as Dreem and Muse-S [105,235], but also frontopolar systems such as Sleep Profiler, which extends the evidence base beyond a small number of consumer headbands [237].

By contrast, cognitive and clinical validations remain more fragmented. Published studies support the feasibility of wearable EEG for ERP recording, drowsiness detection, motor-imagery decoding, and selected neurodegenerative applications, but these studies often use smaller cohorts, task-specific protocols, and device-specific pipelines [25,30,209,227,228,242]. As a result, the validation literature is growing, but it is still uneven across application domains. Published validation evidence is broader than a small set of headband-versus-PSG comparisons. It now includes single-channel A1–A2 and forehead systems, ear-centered EEG, and task-based cognitive validations such as ERP and drowsiness monitoring. Nevertheless, sleep staging remains the most mature and methodologically consistent application, whereas cognitive-state studies more often use smaller cohorts and heterogeneous protocols, which limit direct cross-study comparability [208,209,243].

### 4.5. Summary Table of Validation Studies: Cohorts, Ground Truth, Sensors, and Key Findings

This subsection summarizes key validation studies across device types and application domains. The table compares cohort size or number of recordings/studies, the reference method or ground truth, the sensors used, and the main reported validation metrics or findings. Because studies use different performance indicators, including accuracy, Cohen’s κ, sensitivity, specificity, and AUC, and do not always report all metrics, the reported values should be interpreted in the context of the study design, the application domain, and the classes being evaluated [1,6,227,242]. For consistency of interpretation across studies, Cohen’s κ is treated as a chance-corrected measure of agreement [244], whereas AUC refers to the area under the receiver operating characteristic curve [245].

Although this review does not perform a new quantitative meta-analysis, the validation evidence should be interpreted hierarchically. The strongest and most comparable evidence is available for sleep staging, where wearable EEG systems have been evaluated against PSG in systematic reviews and meta-analyses. These studies indicate moderate-to-substantial agreement overall but also show considerable inter-study variability, which may reflect differences in cohort size, recording conditions, electrode placement, channel count, scoring procedures, and algorithmic pipelines [1,6]. Examples of devices or device classes with relatively stronger PSG-based validation evidence include Muse S, Dreem/Dreem 2, Sleep Profiler/frontopolar EEG, Zmachine single-channel EEG, portable EEG systems evaluated in OSA cohorts, and recent single-lead forehead EEG systems [105,235,236,237,238,243,246,247,248,249].

In contrast, validation studies for cognitive-state tracking, drowsiness, affective-state classification, epilepsy monitoring, and neurodegenerative screening are generally more heterogeneous. They often use smaller cohorts and rely on task-specific labels or comparisons with research-grade EEG rather than PSG [204,208,209,227,228,229,242]. Therefore, reported metrics should not be compared directly across devices unless the reference method, study population, recording environment, and class structure are comparable.

Taken together, wearable EEG systems can capture sleep macro-architecture with moderate-to-substantial agreement relative to PSG, especially for global sleep metrics and coarse sleep-stage classification [1,6]. For cognitive states such as attention, fatigue, and drowsiness, wearable EEG can also distinguish between mental states, although the applied methods and algorithms are not yet well standardized [227,228]. Clinical applications, including epilepsy, neurodegeneration, and mild cognitive impairment, are promising, but they remain largely research-oriented and still require broader clinical validation [240,242]. Overall, meta-analyses and systematic reviews show that wearable EEG performance varies substantially across devices and depends strongly on EEG channel count, algorithmic processing, and the integration of complementary sensors [1,6,227,242].

**Table 7:** Selected EEG wearable validation studies.

Research	Application	Cohort	Reference	Sensors Used	Key Validation Metric	Ref.
Muse S vs. PSG ^1^	Sleep staging	47 56 recruited	Level 1 PSG, trained scorers	EEG ^2^, PPG ^3^, IMU ^4^	κ ^5^ = 0.76, Accuracy 88–96%, Agreement for sleep macro-architecture	[235]
Dreem Headband vs. PSG	Sleep staging	25 31 recruited	PSG + 5 expert consensus	EEG, ACC ^6^, SpO_2_ ^7^	κ = NR, Accuracy 83.5 ± 6.4%	[105]
Sleep Profiler vs. PSG	Sleep staging	47	PSG scored by 5 technologists	Frontopolar EEG	κ = 0.67, after review	[237]
Sleep Profiler vs. PSG	Biomarkers	63	2 nights of self-applied in-home PSG	Frontopolar EEG	75.9%; κ = 0.70; κ = 0.67 (after review); ≥80% epoch agreement (except N1); *p* < 0.0001	[237]
Dreem 2 vs. PSG	Sleep staging in Parkinson’s disease	10	PSG	EEG	Accuracy 55–82%, Useful pattern-level agreement, Some stage overestimation	[236]
EEG analysis	Sleep staging	43	PSG	Varied	κ = 0.68 ± 0.10, Accuracy 78 ± 7%	[6]
Sleep & Alzheimer AI ^8^	Screening/Neuro classification	102	PSG + clinical diagnosis	EEG, ACC (ACM)	κ = NR, Accuracy 90% AUC ^9^ 90%	[240]
Zmachine Z-ALG	Sleep/Wake staging	99	PSG consensus	Single-channel EEG (A1–A2)	κ = 0.85, Sensitivity 95.5%, Specificity 92.5%	[243]
Zmachine Z-PLUS	Sleep staging	99	PSG consensus	Single-channel EEG (A1–A2)	Sensitivity: 84% (N1/N2), 74% (N3), 72% (REM)	[246]
Dry-contact ear-EEG	Sleep staging	20 80 nights	PSG	EEG	κ = 0.73	[247]
Generic ear-EEG at home	Sleep staging	10 20 PSG validations	PSG + trained scorer	EEG with dry electrodes	κ = 0.71	[208]
Portable EEG in OSA ^10^	Sleep staging in OSA	77	Level 1 PSG	EEG, HR, SpO_2_	κ = 0.708; agreement: W 91.7%, N1 55.8%, N2 90.0%, N3 66.6%, REM 85.2%; sensitivity: W 88.2%, N1 54.3%, N2 88.6%, N3 53.9%, REM 84.1%; AUC = 0.897 (AHI ≥ 15), 0.968 (AHI ≥ 30)	[248]
Single-lead forehead device	Sleep staging	106 133 recruited	PSG	Forehead EEG	5-stage κ = 0.70	[249]
Muse ERP validation	Attention/ERP validation	60 + 60 comparisons	Brain Vision ActiChamp recordings	Frontal EEG	N200 Δ = −4.85 μV (95% CI: −5.95 to −3.76; *p* < 0.0001); P300 Δ = 1.37 μV (95% CI: 0.39–2.35; *p* = 0.0069); reward positivity Δ = 2.57 μV (95% CI: 1.58–3.57; *p* < 0.0001)	[13]
e-Glass smart glasses	Epilepsy monitoring/cognitive workload monitoring	24 subjects ~983 h	Research-grade EEG comparison + CHB-MIT dataset/in-house CWM dataset	4-channel dry EEG smart glasses	Average Pearson’s correlation up to 0.93 vs. research-grade EEG; seizure sensitivity 64% with 2.35 false alarms/day; cognitive workload accuracy 74.5%	[204]
GAPses smart glasses	EEG-based biometrics Smart-glasses validation	6	Subject-specific BrainMetrics validation	8-channel dry EEG + EOG smart glasses	Accuracy 99.74%; sensitivity 98.87%; specificity 99.86%; >22 h continuous operation	[205]
Wireless dry ear-EEG	Drowsiness detection	9 35 h	Drowsiness-inducing task labels	EEG	Accuracy: 93.2% (seen), 93.3% (unseen user)	[209]
Around-ear EEG	Affective-state classification	21	Scalp-EEG	8-channel around ear EEG	Accuracy: 67.09 ± 6.14% (arousal), 66.61 ± 6.14% (valence)	[250]
Single-channel in-ear EEG	Emotion monitoring	13	Valence/ arousal protocol	EEG	Accuracy: 72.89% (arousal), 71.07% (valence), 53.72% (4-emotion classification)	[229]

^1^ Polysomnography. ^2^ Electroencephalography. ^3^ Photoplethysmography. ^4^ Inertial measurement unit. ^5^ Cohen’s kappa. ^6^ Accelerometer. ^7^ Peripheral oxygen saturation. ^8^ Artificial intelligence. ^9^ Area under curve. ^10^ Obstructive sleep apnea.

## 5. Discussion and Future Perspectives

In this article, we shed light on smart wearable EEG devices, which represent a dynamically developing research and technology direction and are moving from a controlled laboratory environment towards continuous assessment of brain activity in the real world. They do not measure the same neurophysiological reality as clinical EEG—they provide a reduced, context-dependent, and application-specific representation of brain activity. The overall deviation of wearable EEG from clinical EEG is determined not only by wearability but also depends on electrode coverage, signal quality, and system configuration.

Wearable EEG technologies have progressed from early experimental systems to a wide range of devices for sleep monitoring, cognitive-state assessment, BCI applications, and some emerging clinical uses and early diagnostics. The rapid development of lightweight wearable devices has transformed the accessibility of EEG recording. However, the available evidence also shows that their value depends not only on whether brain activity can be recorded but mainly on the balance between comfort, signal quality, algorithm reliability, and proper validation for the intended use. Across device types, easier setup and better comfort are often achieved at the cost of fewer channels, higher sensitivity to motion and electrode contacts artifacts, and lower spatial resolution than in conventional clinical EEG [1,9,11,163,251]. Despite significant progress in the development of smart wearable EEG technology, challenges remain related to signal quality, reliability, and data interpretability that need to be addressed.

EEG remains a fundamental method for sleep research and real-world neurophysiology due to its ability to directly capture neuronal activity. In sleep monitoring, it is essential for understanding the physiological processes of sleep and its disorders, for distinguishing sleep stages and characterizing sleep architecture, and allows recording slow-wave activity, sleep spindles, and dynamics related to the REM phase. It is a key method for reliable PSG, and even with targeted downsizing and device minimalism, wearable EEG technology is still required for sleep monitoring [212,252]. From a practical point of view, wearable EEG for sleep monitoring is now well established. However, recent meta-analytic evidence indicates that performance remains device-dependent and that agreement with PSG is influenced by electrode configuration, signal-processing pipelines, and validation methodology, emphasizing the importance of standardized evaluation procedures [6]. Several studies and reviews report moderate to substantial agreement with PSG, especially for sleep staging and major sleep parameters. This evidence is strongest for headband, frontopolar, and ear-center systems [1,6,11,18]. This is especially relevant for systems with stable attachment that limit movement during sleep. Direct use for clinical PSG is debatable, because the devices do not meet all requirements. Multimodal recording and the recording of both central and occipital EEG are necessary [253].

In contrast, in everyday neurophysiology, EEG is well suited for continuous assessment of cognitive status, attention, fatigue, and assistive-device control. Wearable devices play a very important role in everyday neurophysiology, as they allow real-time long-term monitoring, which was previously only possible in laboratory conditions. The brain experiences different stimuli during natural activities than in artificial laboratory environments; thus, the devices provide us with valuable information from the field [18,254,255]. Applications in everyday neurophysiology, cognitive-state tracking, and clinical screening are more heterogeneous. Their performance depends strongly on study design, recording environment, target definition, and transparency of the processing pipeline [9,219,227,228]. Smaller devices that retain functionality are now gaining ground in this area. For BCI, neurofeedback or brain performance monitoring, ear EEGs, glasses and headphones with a small number of channels are gaining popularity. They are relatively unobtrusive, and it is interesting for the wearer to embed the EEG in commonly used items without the need for any external device [190,206]. The main question is no longer whether wearable EEG is feasible but under which conditions can it provide reliable and reproducible results.

Although all wearable EEG systems aim to enable unobtrusive neurophysiological monitoring, their performance is strongly influenced by electrode technology, mechanical coupling, and electrode location. Head-worn systems currently provide the best compromise between signal quality, channel count, and multimodal integration, making them the most mature solution for sleep monitoring. Patch-based systems benefit from stable adhesive fixation and low contact impedance but are constrained by very limited spatial information and susceptibility to ocular artifacts. Ear-center devices offer high user comfort and mechanical stability during long-term wear; however, their closely spaced electrodes generally produce lower signal amplitudes than conventional scalp recordings. Glasses-integrated EEG systems achieve the highest level of user acceptance and unobtrusiveness, but their signal quality remains limited by facial muscle activity, blinking, and restricted electrode placement. Consequently, no single form factor currently satisfies all requirements for clinical neurophysiology, sleep monitoring, and continuous real-world use, and device selection remains highly application-dependent (Table 8).

**Table 8:** Comparative overview of major wearable EEG form factors.

Form	Main Advantages	Main Limitations	Typical Channel Count	Sensit. to Artifact	Long-Term Comfort	Multimodal	Validation Status	Primary Applications
Headband	Favorable trade-off between signal quality and usability, mature sleep staging	Susceptible to motion artifacts and dry-electrode pressure discomfort	2–8	Mid	Mid – High	Excellent	Mainly consumer, some systems validated vs. PSG ^1^	Home sleep staging, neurofeedback, BCI ^2^
Patches, Tattoos	Excellent skin contact, discreet and stable	Low channel count, EOG ^3^ contamination, limited spatial coverage	1–2	Low	High	Mid	Some systems have CE/FDA clearance for ambulatory monitoring	Long-term monitoring, epilepsy, sleep screening
Headphone, Earbuds	Excellent comfort and mechanical stability, social acceptability, unobtrusive long-term wear	Lower amplitudes due to non-scalp electrode, limited spatial coverage	1–4	Low – Mid	High	Excellent	Research-grade and consumer-oriented systems	Continuous monitoring, sleep, auditory BCI, neurofeedback, cognitive-state tracking
Glasses	Highly unobtrusive and socially acceptable	Facial muscle artifacts; limited electrode positioning	2–6	High	Very High	Good	Early-stage research and pilot studies	Fatigue monitoring, attention assessment

^1^ Polysomnography. ^2^ Brain–computer interface. ^3^ Electrooculography.

The main limitations of wearable EEG systems remain technical. Compared with conventional clinical EEG, wearable systems usually provide lower signal quality because they use fewer channels and rely on dry or semi-dry electrodes, which are more sensitive to impedance changes, motion, and unstable skin contact [11,20,163,199]. Motion artifacts are a major problem, especially in daytime recordings and in unsupervised home use. Long-term wear also introduces additional problems such as electrode displacement, pressure-related discomfort, sweating, gradual loss of contact quality, and drift in baseline characteristics [11,18,163,186]. These factors reduce robustness and can limit the detection of subtle EEG phenomena, especially when only a small number of channels are available. The spatial placement of EEG electrodes is commonly standardized according to the 10–20 system [256]. However, most wearable EEG devices do not fully implement this system because of their form factor but instead use selected electrode locations, for example, on the forehead or in the temporal region. This leads to reduced spatial coverage and limited neurophysiological interpretability. Individual brain regions can provide different information; for example, during sleep, frontal, central, and occipital electrodes are particularly important for slow-wave activity, sleep spindles, and alpha rhythms, whereas daily neurophysiological applications often require frontal regions for attention and executive functions, as well as central and parietal regions for motor and cognitive processing. For example, the Dreem headband measures at the positions of Fpz, F7, F8, O1, and O2, whereas the Sleep Profiler uses Fpz, AF7, and AF8. To replace the function of clinical PSG, it is necessary to add other physiological sensors and measurements [6,11,106,114,257].

A trend in head-worn EEG systems is a gradual transition from single-modal brain sensing to multi-sensor systems, where EEG acquisition is combined with movement, cardiac, respiratory and peripheral physiological signals. Typical additional sensors are ECG, PPG, accelerometers, temperature sensors, and limited EOG channels. This multimodal integration significantly expands the capabilities of these systems and increases their reliability [11,23,42,228]. Multisensory systems add additional biosignals to compensate for limitations, provide a more comprehensive overview, and increase the robustness of interpretation. This is especially important for sleep monitoring. Neurofeedback, cognitive studies, and epileptiform activity monitoring may not require additional sensors, but such sensors can still improve reliability in these cases. The system’s multimodality is achieved through modular sensor blocks rather than integrated hardware, allowing experiments to be customized to specific needs. The EEG cap g.Nautilus achieves multimodal sensing with external sensor connections through additional inputs [258]. Similarly, OpenBCI Ultracortex uses a Cyton board as a basic biosensor platform capable of acquiring additional bioelectrical signals (EMG, ECG) [259]. A multimodal wearable EEG device represents an important advance because it allows the simultaneous recording of multiple physiological signals. For example, OpenBCI Galea is a real multimodal platform that combines EEG with EOG, EMG, EDA, and PPG within a single synchronized hardware architecture [42]. Multimodal analysis is particularly relevant for applications such as affective computing, cognitive load assessment, and BCIs.

However, the benefits of multimodal sensing can only be fully exploited if the underlying signals are acquired with sufficient resolution. The sampling rate determines the resolution not only of EEG signals but also affects the ability to accurately capture and interpret physiologically relevant neural dynamics. The requirements for the sampling rate are determined by the frequency needed to record the measured signal and the needs of the targeted application. According to the Nyquist–Shannon sampling theorem, the sampling rate must be at least twice the highest frequency component of the signal to ensure accurate reconstruction, so for EEG, 250 Hz is the most common sampling rate for sleep monitoring and neurofeedback [211,212,213,260]. For more demanding applications such as BCI or research, a higher sampling rate, often 500 Hz, is also suitable. Additional sensors may have different requirements; for example, EMG requires a sampling rate of around 1000 Hz [261]. ECG operates in a similar sampling range (250–500 Hz), and PPG or respiration are recorded at lower sampling rates due to their slower dynamics. Over-sampling increases power consumption without a significant increase in information content, while under-sampling risks losing critical information, especially for high-frequency signals such as EMG [262].

Another important challenge is the need for sophisticated signal processing. Because wearable EEG often operates with limited and noisy data, reliable analysis depends on careful preprocessing, artifact reduction, quality control, and well-designed classification algorithms [188,189,191]. Recent reviews of EEG preprocessing and feature extraction further highlight that variability in artifact removal strategies and feature engineering remains a major source of inconsistency in automated sleep staging performance across studies [263]. At the same time, complex algorithms do not fully solve the hardware limitations. Good performance may still depend on proper device placement, user compliance, and training models for a specific task [1,11,188,189]. In addition, comparisons across studies remain difficult because devices differ in electrode positions, reference schemes, validation protocols, and reported performance metrics [6,11,188]. In this review, the term signal quality is therefore not treated as a single device property. Depending on the application, it may refer to low noise or high signal-to-noise ratio, stable electrode–skin contact, agreement with conventional EEG or PSG, robustness against motion and physiological artifacts, detectability of relevant EEG features such as ERP components or sleep spindles, sleep-staging performance, or reliability across repeated recordings. For this reason, qualitative terms such as good, excellent, or high-quality signal should be interpreted only in relation to the specific metric, validation setting, and application domain reported in the cited study. Key validation dimensions and commonly reported performance metrics are summarized in Table 9.

**Table 9:** Key validation dimensions for wearable EEG systems.

Validation Dimension	Typical Metrics	Clinical and Technical Relevance
Signal quality	SNR ^1^, contact impedance, spectral noise floor	Determines physiological signal fidelity
Agreement with conventional EEG ^2^	Correlation coefficient, coherence, RMSE ^3^	Assesses comparability with scalp EEG
Sleep staging performance	Accuracy, Cohen’s κ, F1-score	Evaluates sleep-monitoring reliability
Artifact robustness	Percentage of usable data, artifact rejection rate	Reflects real-world applicability
ERP ^4^ detectability	Amplitude error, latency error	Important for cognitive and BCI ^5^ applications
Long-term reliability	ICC ^6^, test–retest correlation	Supports longitudinal monitoring
PSG ^7^ agreement	Bland–Altman analysis, TST ^8^, SOL ^9^, WASO ^10^ agreement	Clinical benchmark for sleep assessment

^1^ Signal–noise ratio. ^2^ Electroencephalography. ^3^ Root mean square. ^4^ Event-related potential. ^5^ Brain–computer interface. ^6^ Intraclass correlation coefficient. ^7^ Polysomnography. ^8^ Total sleep time. ^9^ Sleep onset latency. ^10^ Wake after sleep onset.

Edge computing may help solve part of this problem. It can support onboard feature extraction, artifact reduction, and lightweight sleep staging while reducing the amount of transmitted data [232,233]. However, embedded processing also increases computational load and may shorten battery life. For this reason, future wearable EEG systems will likely use hybrid or hierarchical architectures that combine edge inference with cloud-based analysis [232,233,234]. Beyond computational efficiency, future wearable EEG systems must also address practical challenges associated with long-term deployment. Data privacy and cybersecurity are becoming increasingly important as wearable EEG platforms rely on wireless communication, cloud-based storage, and AI-driven analytics. Because EEG signals may contain highly individualized physiological signatures, future systems will require secure transmission, encryption, anonymization procedures, and compliance with regulatory frameworks such as GDPR and HIPAA. Another important research direction is the implementation of real-time edge processing. While cloud-based analysis enables computationally demanding algorithms, reliance on continuous connectivity increases latency, power consumption, and privacy concerns. Future architecture will likely combine lightweight onboard processing with cloud-assisted analysis to balance computational efficiency and user autonomy. Finally, broader adoption will require internationally accepted benchmarking datasets, harmonized validation protocols, and transparent reporting standards that allow objective comparison across devices and studies.

Higher sampling rates, a higher number of channels, multimodality, wireless signal transmission, and signal processing all affect battery life. Power supply is also one of the challenges in the design and performance of wearable devices and varies depending on the architecture and intended use of the device. For sleep measurement, the device needs to last the whole night, and for daytime neurophysiology, it should operate at least throughout the day without the battery becoming a burden during wear. Headbands have the best durability, also because they have fewer channels than caps and are still relatively large for battery placement (usually 8–12 h, Sleep Profiler even 30 h). EEG caps have the worst durability, because they have many channels and high sampling rates (2–10 h). Headsets last about 8–12 h, and tattoos are questionable because they often have the battery out and are generally still in the early stages of use. Interestingly, patches that last 8–12 h for a small number of channels are promising for long-term monitoring and clinical use. Glasses have limited space in the frames for the battery, so they last around 4–10 h. The battery life can be extended by using fewer channels, edge processing, and the use of low-power chips. However, there is ongoing research into low-power alternatives to minimize battery footprints in devices. Energy harvesting in wearable EEG systems could be an interesting option, especially for the smallest devices such as patches and tattoos, and refers to the use of ambient energy sources, such as body motion, heat, or electromagnetic fields, to partially or completely power the device. This could reduce dependence on conventional batteries [263,264].

These challenges define several actionable research directions for future wearable EEG systems. First, future devices should be evaluated using standardized protocols that report signal quality, artifact robustness, sleep-stage agreement, long-term reliability, and performance under real-world recording conditions. Second, battery-aware system design should combine adaptive sampling, low-power electronics, selective sensor activation, and hybrid edge–cloud processing to support overnight and daytime recordings. Third, privacy-preserving architectures should reduce unnecessary raw EEG transmission and support secure local preprocessing where possible. Finally, real-time applications such as neurofeedback, sleep modulation, and closed-loop monitoring require low-latency edge algorithms that are validated not only in controlled laboratory settings, but also during unsupervised home or everyday use [232,233,234,264,265].

Regulatory approval profiles remain highly heterogeneous across wearable EEG domains, as most presented devices are intended for consumer purposes or research rather than formal clinical use. Most market-available sleep headbands, smart glasses, and integrated earbuds are legally classified as low-risk general wellness devices under current FDA and CE medical frameworks. When focusing on medical approvals, devices designed for ordinary consumers typically lack them or possess them strictly for wellness applications. For example, the MUSE system has obtained FDA clearance but not as a certified medical device. While these consumer systems frequently undergo rigorous technical validation against conventional EEG or polysomnography, often demonstrating high correlation coefficients above 0.85 or robust Cohen’s kappa values above 0.70 for sleep staging, this technical validation does not automatically translate to regulatory clearance for clinical decision support.

In contrast, a limited subset of wearable devices is clinically certified for direct use in clinical settings. For instance, the Advanced wearable EEG headset has achieved CE and FDA clearance, the Dreem headband holds FDA clinical approval, the GTec cap Nautilus features both CE and FDA clinical approvals, and the Neurosteer patch is similarly FDA clinically approved [266,267,268]. Traditional EEG caps often secure FDA and CE clearances because they are tailored for standard clinical environments, while specific patches receive approvals to detect critical conditions such as epileptic seizures. Formal regulatory approval is not required for classic lifestyle sleep monitoring or daily neurophysiology. However, for disease diagnosis, clinical decision-making, treatment guidance, or active patient monitoring and treatment, these rigorous approvals are necessary.

Therefore, regulatory status should not be confused with technical performance. Much research-grade wearable EEG systems demonstrate promising agreement with conventional EEG or polysomnography, while only a limited subset has completed the regulatory pathways required for clinical diagnosis, treatment guidance, or medical decision support. Medical-grade systems, such as select clinical patches or diagnostic forehead bands, must explicitly obtain formal regulatory pathways, including FDA 510(k) clearance or EU Medical Device Regulation certification. This transition demands strict compliance with medical software standards like IEC 62304 [269], biocompatibility testing for long-term skin contact under ISO 10993, and verified clinical efficacy in multi-center clinical trials. Future literature and studies must therefore meticulously maintain a clear boundary between research-grade validation, consumer deployment, and formally cleared clinical instrumentation to ensure safe and compliant adoption.

These observations have important implications for future development. Further progress will likely depend on innovative electrode materials and more stable and comfortable device designs. Recent meta-analytic findings also highlight the need for harmonized benchmarking datasets, standardized validation frameworks, and transparent reporting of sleep-staging metrics to improve comparability across wearable EEG platforms [6]. It will also depend on stronger multimodal integration and adaptive algorithms that can handle differences between users and changes in the signal over time [11,18,163,270]. Future wearable EEG systems may combine EEG with motion, cardiovascular, respiratory, or other biosignals to improve context awareness and artifact interpretation [11,187,271]. However, technical progress alone will not be sufficient. For wider clinical use, larger validation studies, clearer evaluation methods, and more reproducible results will be needed [1,6,217,219,220]. In addition, if wearable EEG systems are used for screening, remote monitoring, authentication or decision support in healthcare, regulatory requirements and data protection must also be addressed.

## 6. Conclusions

In this review, we focused on non-invasive, easy-to-use devices. Wearable EEG devices cover a wide range of designs, from minimal, almost invisible devices to full-fledged multi-channel EEG caps. Each group has its advantages and disadvantages and offers different levels of signal quality, spatial coverage, and comfort, with different suitability for daily neurophysiology and sleep monitoring applications. While sleep monitoring requires comfort and long-term stability, everyday neurophysiology focuses on the ability to capture neural signals during natural activities such as work, study, or BCI. In this context, wearable EEG devices must tolerate motion artifacts.

The choice of a wearable EEG system depends on the target application and wearer-specific constraints. Head-worn systems provide broader spatial coverage and remain closest to conventional EEG, whereas headbands, patches, and tattoo-like systems prioritize comfort and long-term stability. For sleep monitoring, designs that minimize sleep disturbance while maintaining stable skin or electrode contact are particularly relevant. In-ear, headphone-based, and glasses-integrated systems extend EEG monitoring toward more unobtrusive everyday use, although they provide more limited spatial coverage.

At the same time, hardware alone does not determine the usefulness of wearable EEG. Signal quality, artifact handling, automated analysis, and multimodal integration are equally important. This multimodal approach improves artifact suppression and allows for more robust inference of cognitive and physiological states in naturalistic environments. Advances in materials science are redefining the electrode–skin interface. Textile-based electrodes, graphene coatings, stretchable conductive composites, and bioinspired microneedle arrays help to build comfortable, lower-impedance devices, while the incorporation of flexible electronics and thin-film integration leads to nearly invisible EEG systems embedded in everyday clothing or accessories.

Recent progress in machine learning has improved automated sleep staging and supported progress in cognitive-state detection and other EEG-based applications. Combining EEG with motion, cardiovascular, respiratory, or eye-related signals can improve robustness and interpretability. Edge and hybrid edge–cloud processing also supports more practical real-time operation in wearable systems.

Despite this progress, important challenges remain. Wearable EEG devices still differ widely in electrode design, channel count, signal quality, and validation strategy. For broader clinical and real-world use, larger validation studies, better standardization of evaluation methods, and more transparent benchmarking are still needed. Personalization is also likely to become increasingly important, because EEG patterns and signal quality can vary substantially between users and across recording conditions.

The value of wearable EEG lies not only in measurement accuracy, but also in its ability to provide a continuous real-time view of brain states and support the use of these data in real-world contexts. It should also be noted that relatively few application areas are currently available. Several devices currently offer meditation or neurofeedback applications for home well-being, but this field still has substantial room for development, and future systems may support broader applications for cognitive training and brain-based environmental control. Open SDKs offered by some companies may support the development of new application-specific wearable EEG tools.

Overall, wearable EEG is moving toward more accessible and user-centered neurophysiology. By reducing the gap between laboratory recording and real-world monitoring, these technologies may support home sleep assessment, long-term brain monitoring, early screening, and future digital-health applications. Continued progress in materials, signal processing, multimodal sensing, and adaptive algorithms will likely make wearable EEG an increasingly important part of practical neuroscience and personalized healthcare.

Future wearable EEG devices are likely to become more multimodal and easier to use, with less need for additional user manipulation. Overall, integrating additional sensors with EEG is increasingly important because complementary signals can improve robustness, support artifact and noise reduction, and provide a broader view of the user’s physiological state. In the future, wearable EEG has the potential to become a gateway to accessible neurophysiology. By reducing the barrier between laboratory neuroscience and the real-world environment, these technologies can enable continuous brain monitoring, preventive neurological screening, digital therapy, and remote clinical assessment. In wearable EEG, signal interpretation is increasingly algorithm-driven, with artificial intelligence playing a key role in compensating for hardware limitations and enabling real-world applications. An optimal wearable EEG device will not necessarily maximize channel count or sampling rate but should provide an application-specific balance between signal quality, comfort, battery life, and reliable interpretation of the recorded data.

## Data Availability

No new data were created or analyzed in this study.

## Abbreviations

The following abbreviations are used in this manuscript:

AI	Artificial intelligence
API	Application programming interface
AR	Augmented reality
ASR	Artifact subspace reconstruction
AUC	Area under curve
BCI	Brain–computer interface
BLE	Bluetooth low energy
ECG	Electrocardiography
EDA	Electrodermal activity
EEG	Electroencephalography
EMG	Electromyography
EOG	Electrooculography
ERP	Event-related potential
fNIRS	Functional near-infrared spectroscopy
GSR	Galvanic-skin response
HR	Heart rate
HRV	Heart rate variability
ICA	Independent component analysis
ICU	Intensive care units
IMU	Inertial measurement unit
MCI	Mild cognitive impairment
PPG	Photoplethysmography
PSG	Polysomnography
REM	Rapid eye movement
RESP	Respiration
SDK	Software development kit
SNR	Signal-to-noise ratio
SpO_2_	Peripheral oxygen saturation
SSVEP	Steady-state visually evoked potential
TEMP	Temperature
VR	Virtual reality
